# Targeting the EpCAM‐AXL axis to overcome drug resistance in lung cancer

**DOI:** 10.1002/1878-0261.70342

**Published:** 2026-10-06

**Authors:** Alexa Guerrero‐Alba, Mark Shacker, Timothy P. Fleming, Michael A. Smith, Ross M. Bremner, Narendra V. Sankpal

**Affiliations:** ^1^ Norton Thoracic Institute St. Joseph's Hospital and Medical Center Phoenix AZ USA; ^2^ School of Medicine Creighton University, Phoenix Health Sciences Campus AZ USA

**Keywords:** AXL, drug resistance, EpCAM, proteasome

## Abstract

Drug resistance significantly limits the long‐term effectiveness of targeted therapies in lung cancer. AXL, a receptor tyrosine kinase (RTK), is a well‐established driver of drug resistance in many cancers, including lung cancer. Epithelial cell adhesion molecule (EpCAM), which is commonly overexpressed in epithelial cancers, is also a critical signaling molecule. In this study, we elucidated the role of EpCAM in drug resistance and its mechanistic association with AXL. We found that soluble epithelial cell adhesion molecule (sEpCAM) expression robustly enhanced hallmark cancer phenotypes and promoted pro‐survival functions by upregulating AXL expression. Gene expression and pathway analyses further identified significant alterations in core cancer signaling pathways, including NF‐kB, STAT3, AKT, and ERK. The ectopic expression of sEpCAM concurrently upregulated endogenous EpCAM expression in the tumor environment. Functionally, sEpCAM confers broad resistance to conventional chemotherapy. Drug sensitivity assays revealed that proteasome inhibition effectively reversed the sEpCAM‐induced drug resistance by reducing AXL protein levels. Translational analysis of the lung cancer patient cohort data indicated that patients with aggressive disease co‐expressing EpCAM and AXL may be particularly amenable to proteasome‐based therapy. Characterization of the EpCAM‐AXL axis gene signature further uncovered connections to p53, integrin, and Rho GTPase signaling as well as an intriguing link to tumor immune evasion. Our findings suggest that EpCAM‐AXL co‐expression in a subset of patients with lung cancer serves as both a predictive biomarker and a promising therapeutic target to counteract treatment failure.

## Graphical abstract

Lung cancer cells often evade therapy by hijacking signaling pathways. We reveal that cleaved EpCAM (sEpCAM) stabilizes the oncogenic protein AXL, driving NF‐κB and STAT3‐mediated chemoresistance. This EpCAM‐AXL axis identifies a high‐risk patient subset with poor prognosis. Notably, proteasome‐mediated degradation of AXL disrupts this protective mechanism, restoring therapeutic sensitivity and offering a potential strategy to overcome treatment failure.

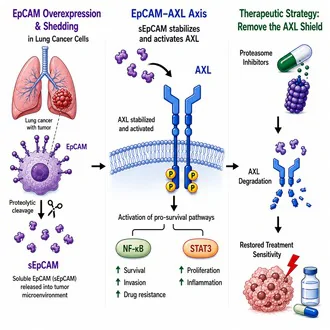

**Abbreviations**
DMEM: Dulbecco's modified Eagle's medium
EGF: Epidermal Growth Factor
EMT: Epithelial‐to‐mesenchymal transition
EpCAM: Epithelial Cell Adhesion Molecule
GAS6: Growth Arrest‐Specific 6
LUAD: Lung Adenocarcinoma
NSCLC: Non‐small cell lung cancer
OS: Overall survival
PBS: phosphate‐buffered saline
PDX: Patient‐derived xenografts
qRT‐PCR: Quantitative Real‐Time PCR
RIPA: Radioimmunoprecipitation assay buffer
RTK: Receptor tyrosine kinase
TCGA: the cancer genome atlas
TGF‐β: Transforming Growth Factor Beta
UPS: Ubiquitin‐proteasome system

## 1 Introduction

Non‐small cell lung cancer (NSCLC) accounts for approximately 85% of all lung cancer cases and is a leading cause of cancer‐related mortality worldwide. It is projected that 2 041 910 new cancer cases and 618 120 cancer deaths will occur in the United States in 2025 [1]. The 5‐year overall survival rate of patients with metastatic NSCLC is less than 5%, highlighting the urgent need for more effective therapies and strategies to overcome treatment failure. Mutations in oncogenic drivers such as EGFR, KRAS, ALK, BRAF, and MET are critical for tumor initiation, progression, and frequently mediate resistance to targeted therapies. For example, a significant clinical challenge in NSCLC is the development of acquired resistance to systemic therapies, which occurs in approximately 30% of all EGFR‐mutated NSCLC cases [2]. Established mechanisms of resistance include secondary EGFR mutations, MET amplification, and activation of alternative receptor tyrosine kinases [3, 4, 5]. Similarly, chemotherapy resistance is driven by diverse molecular and cellular adaptations [6, 7]. TAM receptor (TYRO3, AXL, and MerTK) family has emerged as a key mediator of therapeutic failure in NSCLC [8]. Preclinical studies have indicated that AXL inhibition can restore sensitivity to EGFR inhibitors, establishing AXL as a promising therapeutic target [3, 9]. AXL is frequently overexpressed in drug‐resistant tumor cells and is strongly associated with epithelial‐to‐mesenchymal transition (EMT). AXL also promotes progression by this fundamental biological process, which involves the loss of epithelial traits and acquisition of mesenchymal properties, which in turn enhances cellular invasiveness, metastatic potential, and resistance to both chemotherapy and targeted agents [10, 11]. AXL is functionally integrated with RTKs such as EGFR, HER2, HER3, and MET, frequently influencing their activation [12]. When activated by its ligand GAS6 or through interactions with HER family receptors, AXL initiates critical downstream signaling cascades, including PI3K/AKT, NF‐κB, STAT3, and MAPKs, ultimately driving cell survival, proliferation, and resistance to apoptosis [8].

In addition to direct RTK signaling, membrane‐bound proteases play a critical role in cancer progression by modulating receptor activity. ADAM17 (A disintegrin and metalloproteinase‐17), a key membrane protease, proteolytically cleaves the ectodomains of numerous transmembrane receptors and ligands such as TGFα, amphiregulin, betacellulin, and TNFα, thereby influencing cell–cell communication, proliferation, inflammation, metastasis, and drug resistance [13, 14, 15]. ADAM17, which is overexpressed in NSCLC, also liberates the EpCAM ectodomain (sEpCAM), enabling release of the EpCAM intracellular domain to promote nuclear signaling [16]. EpCAM expression in tumor cells is often linked to larger tumor size, nodal metastasis, and dedifferentiation in some cohorts [17, 18]. In squamous cell carcinoma of the lung, high EpCAM expression correlates with an advanced stage, poor differentiation, and shorter survival [19, 20]. EpCAM has also been implicated in activation of the PI3K/AKT/mTOR pathway [21, 22]. Our previous work demonstrated that EpCAM ablation in RAS‐transformed cells reduced the tumor burden via suppression of JNK and NF‐κB signaling [23, 24]. In addition, our recent molecular modeling study suggests that somatic mutations constitute up to 5%, which disrupt EpCAM structure–function and provide a survival advantage [25]. EpCAM expression decreases or is lost during EMT, crucial for metastasis, only to potentially reappear at metastatic sites. Heterogeneous expression patterns and EMT‐associated downregulation introduce complexities, leading to conflicting reports correlating with disease progression or outcome [20, 25, 26, 27, 28, 29]. Despite its known pro‐tumorigenic role, the precise contribution of EpCAM, particularly its soluble form, to acquired drug resistance remains largely unexplored [30, 31].

This study aimed to define the role of EpCAM in promoting drug resistance. Our primary focus was to elucidate the mechanistic link between EpCAM expression, particularly its soluble form (sEpCAM), and AXL activation. We hypothesized that this interaction forms a novel EpCAM‐AXL axis that could contribute to drug resistance. Through a comprehensive strategy encompassing molecular biology, functional assays, and translational analysis of patient data, we propose that the EpCAM‐AXL axis represents a potential actionable vulnerability. Specifically, the co‐expression of EpCAM and AXL identifies a therapeutic opportunity within a subset of non‐small cell lung cancer patients. This finding strongly supports investigating targeted interventions of proteasome inhibition.

## 2 Materials and methods

### 2.1 Cell culture and reagents

The human non‐small cell lung cancer (NSCLC) cell lines A549 (RRID:CVCL_0023), NCI‐H1299 (CVCL_0060), NCI‐H1975 (CVCL_1511) and viral packaging cell lines Phoenix‐Ampho (CVCL_H716) and HEK‐293T (CVCL_0063) were procured from ATCC and maintained in appropriate growth media (DMEM or RPMI‐1640; HyClone, Logan, UT, USA) supplemented with 10% FBS and 1% penicillin–streptomycin in a humidified incubator at 37 °C with 5% CO_2_. Cell lines were authenticated by short tandem repeat (STR) profiling within the last 3 years. The cell lines used were mycoplasma‐free as verified by regular testing.

Recombinant human GAS6 (#885‐GSB; R&D Systems, Minneapolis, MN, USA), EGF (#AF‐100‐15; PeproTech, Cranbury, NJ, USA), and TGF‐β (#100‐21; PeproTech) were reconstituted and used, as described in the Results section.

### 2.2 Generation of EpCAM variant stable cell lines

The coding sequences for full‐length human EpCAM (EpCAM‐314F, GenBank ID: NM_002354) and its deletion mutants (EpCAM‐288F, EpCAM‐265F, and EpCAM‐140F) were subcloned into the pBABE‐Puro or pBABE‐Hygro retroviral expression vectors, as previously described [32]. To generate replication‐incompetent retroviral particles, near‐confluent Phoenix‐AMPHO packaging cells in a six‐well plate were transfected with 2.5 μg of pBABE‐Puro‐EpCAM construct/s using FuGENE‐HD (#E2311; Promega, Madison, WI, USA). Viral supernatants were harvested 48 h post‐transfection, sterile‐filtered through 0.45 μm membranes, and added to A549 cells supplemented with 8 μg·mL^−1^ protamine sulfate. The cells were allowed to recover for 48 h before antibiotic selection.

### 2.3 Growth factor and drug treatments

A549 cells expressing EpCAM variants were treated with EGF (25 ng·mL^−1^) or TGF‐β (5 ng·mL^−1^) for 24 h before western blotting. For GAS6 stimulation and pathway analysis, cells were serum‐starved overnight (2% serum) and then treated with recombinant GAS6 (200 ng·mL^−1^) for 30 min. All the drug compounds were obtained from Selleck Chemicals (The Woodlands, TX, USA), Sigma‐Aldrich (St. Louis, MO, USA), or LC Laboratories (Woburn, MA, USA) (Table S1). The IC50 values for A549 cells were determined based on previous reports and data from the Genomics of Drug Sensitivity in Cancer (GDSC) Portal (https://www.cancerrxgene.org/). Collagen (Enzo #ALX‐522‐435) and fibronectin (#33016015; ThermoFisher) were used according to the manufacturer's recommendations. This solution was then added to the plates, incubated at room temperature for 1–2 h (or 4 °C overnight), and subsequently washed 2–3 times with sterile 1X PBS. Fibronectin coating was performed at 5 μg·cm^−2^ by diluting the stock in sterile 1X PBS. This solution was added to the plates, incubated at room temperature for 30–60 min, and washed 1–2 times with sterile 1X PBS.

### 2.4 Targeting EpCAM and AXL via lentiviral shRNA

AXL shRNA, along with a non‐targeting scrambled control shRNA, was obtained as plasmid constructs from Sigma‐Aldrich (NM_001699, TRCN0000000572). Lentiviral particles were produced by co‐transfecting each shRNA plasmid with the second‐generation packaging plasmid pLKO.1 (shGFP or shAXL) into 293T cells using Fugene‐HD, as previously described [29]. Briefly, supernatants containing viral particles were harvested, filtered through 0.45 μm membranes, and transduced in medium supplemented with 8 μg·mL^−1^ polybrene. Specific gene knockdown was confirmed using quantitative real‐time PCR (qPCR) to assess the mRNA expression levels and western blot analysis. For rescue experiments, pCDNA‐GFP or pCMV‐AXL was transiently transfected into A549‐shEpCAM‐2 cells.

### 2.5 Establishment of matched *in vivo* and *in vitro* cell models

All animal procedures were performed in strict accordance with the guidelines established by the St. Joseph Hospital and Medical Center Institutional Animal Care and Use Committee (Animal Welfare Assurance # A3519‐01 Protocol: 616). Experiments utilized 4‐6‐week‐old female athymic nude mice, strain Crl : NU‐Foxn1^nu^ (Charles River Laboratories, Wilmington, MA; strain code: 088).

Mice were housed in a specific pathogen‐free facility (SPF). Animals were maintained in temperature‐controlled (22 ± 2 °C) and humidity‐controlled (50 ± 10%) rooms on a 12‐h light/dark cycle. Mice were provided with standard rodent chow and autoclaved water *ad libitum*. Animals were monitored daily for signs of distress, and body weight was recorded weekly. All experimental procedures, including subcutaneous injections and tumor harvesting, were conducted by trained personnel using aseptic techniques within a biosafety cabinet to maintain the SPF status of the colony. To generate tumor‐derived cells, 5 × 10^6^ A549, H1299, or H1975 (wild‐type or GFP‐Puro) cells were implanted subcutaneously into the flanks of the mice. Following a four‐week engraftment period, tumor xenografts were harvested, mechanically dissociated, and enzymatically digested to isolate viable tumor‐derived cells. All cell lines, including *in vitro* parental controls and newly derived tumor cells, were routinely screened for Mycoplasma contamination. To maintain a sterile environment and prevent bacterial contamination, cultures were supplemented with 10 μg·mL^−1^ ciprofloxacin. Experiments were performed on cells within passages 5–8 to ensure phenotypic consistency and reproducibility.

### 2.6 Quantitative real‐time PCR (qRT‐PCR)

Total RNA was extracted using TRIzol (#15596026; ThermoFisher) according to the manufacturer's instructions. cDNA was synthesized using a High‐Capacity Reverse Transcription Kit (#4368814; ThermoFisher Scientific). qRT‐PCR was performed using gene‐specific primers for EpCAM, E‐cadherin, Vimentin, AXL, and MAPK13 supplemented with Fast SYBR Green Master Mix (#4385612; Thermo Scientific) on a Step One Plus Real‐Time PCR system (Applied Biosystems). Relative gene expression was calculated using the $2^{-\Delta \Delta C_{t}}$ method and normalized to the housekeeping gene 18SrRNA. Relative expression fold‐change, determined by ΔΔ*C*_t_, compared tumor‐derived cells to 2D cultured cells. Primer sequences are listed in Table S2.

### 2.7 AXL immunoprecipitation and ubiquitin blotting

After a brief wash with cold PBS, the cells were lysed in ice‐cold lysis buffer (50 mm Tris–HCl pH 8, 150 mm NaCl, 1% Triton‐X100, 0.1% SDS, and 5% glycerol) supplemented with protease inhibitor cocktail and 1‐mm Sodium Ortho‐vanadate (#11836170001; Sigma). The cell lysates were cleared by centrifugation, and the protein concentration was determined. Equal amounts of protein (200 μg) were incubated overnight at 4 °C with 2 μg AXL antibody. Protein A/G agarose beads (#20421; ThermoFisher) were added for 2 h at 4 °C to capture the immune complexes. Beads were washed extensively with lysis buffer and boiled in SDS/PAGE loading buffer to elute immunoprecipitated proteins. The samples were resolved by SDS/PAGE, transferred to a PVDF membrane, and probed with an anti‐ubiquitin antibody, followed by HRP‐conjugated secondary antibodies.

### 2.8 Conditioned media collection to assess sEpCAM

Transiently transfected HEK293 or stable A549 cells were incubated in Opti‐MEM (#31985070; ThermoFisher) serum‐free medium for 48 h. Conditioned media were collected after 48 h, cleared, and concentrated to 5X using a 3‐KDa MWCO Amicon Ultra Centrifugal Filter (#UFC5003; Millipore‐Sigma, Burlington, MA, USA).

### 2.9 Western blotting

The cells were washed with cold PBS, followed by lysis in RIPA buffer (#9806; Cell Signaling Technology, Danvers, MA, USA) supplemented with a protease inhibitor cocktail (#11836170001; Sigma) and 1 mm sodium orthovanadate. Protein concentrations were determined using Bicinchoninic acid assay (#23227; Thermo Scientific). Equal amounts of protein were resolved on NuPAGE‐Bis‐Tris Gels (ThermoFisher) and transferred to PVDF membranes (#10600029; Cytiva, Marlborough, MA, USA). Membranes were blocked with 5% non‐fat dry milk (#9999; Cell Signaling Technology) and incubated with primary antibodies, followed by incubation with HRP‐conjugated secondary antibodies (Cell Signaling Technology). Protein bands were visualized using a chemiluminescence imager (Odyssey Fc, Li‐COR). GAPDH or β‐actin served as loading controls. Protein band density was determined using the image studio V5.2 software. The antibodies used for western blotting included anti‐FLAG, anti‐EpCAM, anti‐AXL, anti‐p‐AXL, anti‐p‐AKT, anti‐AKT, anti‐p‐ERK, anti‐ERK, anti‐E‐cadherin, anti‐vimentin, anti‐c‐Met, anti‐p53, anti‐EGFR, anti‐p‐EGFR, anti‐STAT3, anti‐GAPDH, and anti‐β‐actin (Table S3).

### 2.10 Cell invasion, migration, viability, and proliferation assay

Cell invasion and migration were assessed using 24‐well Transwell inserts (8 μm pore size). For invasion assays, inserts were pre‐coated with a Matrigel basement membrane matrix (Cat. No. 354480; Corning, NY, USA). Uncoated inserts were used for migration assays. A total of 5 × 10^4^ cells were suspended in 200 μL serum‐free medium and seeded into the upper chamber of the Transwell inserts. The lower chamber of each well was filled with 600 μL of complete growth medium containing 10% fetal bovine serum as a chemoattractant. After 48 h of incubation at 37 °C in a humidified 5% CO_2_ incubator, non‐invading or non‐migratory cells on the top surface of the membrane were removed using a cotton swab. Cells that migrated to the lower surface of the membrane were fixed with 4% paraformaldehyde for 15 min and stained with 0.1% crystal violet for 10 min. After several washes with deionized water, the membranes were air‐dried. The number of invading or migrating cells was quantified by counting the cells from at least five random fields per well under a light microscope. For the viability assay, after 48–72 h of drug treatment, the culture medium was aspirated and gently washed once with sterile PBS. The cells were then fixed with 4% paraformaldehyde for 15 min at room temperature and stained with 0.1% crystal violet solution for 10 min. The unbound stain was removed by repeated gentle washing with deionized water. Plates were air‐dried at room temperature and imaged.

For the cell viability assay under nutrient restriction, subconfluent A549 cells were cultured in 2% fetal bovine serum (FBS) for 72 h at 37 °C in a humidified 5% CO_2_ incubator. The cells were then washed with PBS and stained with 0.1% crystal violet for 10 min. After several washes with deionized water to remove unbound stain, the membranes were air‐dried and imaged. For the proliferation assay, cells were seeded in 96‐well plates at a density of 2000 cells/well in triplicate and allowed to adhere for 24 h. Subsequently, the cells were treated with various concentrations of drugs or vehicle for 72 h. Cell growth was assessed using the CellTiter‐Glo Luminescent Cell Viability Assay (#G7570; Promega), according to the manufacturer's instructions. Data were normalized to vehicle‐treated controls and expressed as a percentage of control proliferation.

### 2.11 Wound‐healing assay

A549 cells were seeded in 12‐well plates and grown to confluency. A sterile 200 μL pipette tip was used to create a uniform scratch across the cell monolayer. Free‐floating cells were removed by gentle washing with PBS and grown in a fresh 5% serum medium. After 48 h, cells were washed twice with PBS, stained with 0.2% crystal violet for 5 min, washed with PBS, and imaged.

### 2.12 Luciferase reporter assays

The reporter assay was performed as previously described [24]. Briefly, cells were cultured in a 12‐well plate for 24 h. DNA constructs 400 ng‐SRE‐Luc (E1340; Promega), NF‐κB (#E8491; Promega), Myc, STAT3 (# PT3535‐5; Promega), and the internal control plasmid 100 ng‐pRLSV40‐Luc (#E2231; Promega) were transfected using FuGENE‐HD (#E2311; Promega). After 12 h of transfection, cells were serum‐starved for 12 h and treated with 20% FBS overnight. Cells were lysed, and the dual‐luciferase assay was performed using the Dual‐Luciferase Reporter Assay System (#E1910; Promega). The mean values of luciferase activity relative to the untreated cells were calculated from triplicate wells. The experiments were repeated three times to ensure consistency. Data are presented as the mean ± SD (standard deviation) of three technical replicates. *P < 0.05, **P < 0.001, two‐tailed Student's *t*‐test.

### 2.13 Patient TCGA cohorts, survival, and bioinformatics analysis

Gene expression and clinical survival data for lung squamous cell carcinoma (LUSC) and lung adenocarcinoma (LUAD) patients were obtained from The Cancer Genome Atlas (TCGA) public databases (https://portal.gdc.cancer.gov/ and Xena Browser https://xenabrowser.net/). Patients were dichotomized into high and low expression groups based on the median expression levels of EpCAM or AXL. Overall survival (OS) curves were analyzed using the Kaplan–Meier method, and the log‐rank test was used to assess the statistical significance between survival curves. Hazard ratios (HRs) and 95% confidence intervals (CIs) were calculated to evaluate their prognostic value. Gene Expression Omnibus (GEO) datasets (GSE30219, GSE31210, GSE43580, GSE32989, GSE50081, and GSE5949) were acquired from https://www.ncbi.nlm.nih.gov/gds and analyzed for hierarchical clustering and heat map visualization using the GENE‐E tool (https://software.broadinstitute.org/GENE‐E/). To elucidate the functional implications of the EpCAM‐AXL gene signature, Gene Ontology (GO) enrichment analysis was conducted using Gene Ontology Server (https://geneontology.org/). This analysis aimed to identify enriched biological processes, molecular functions, and cellular components associated with these genes.

### 2.14 Statistical analysis

All experiments were performed with a minimum of three independent biological replicates. Data are presented as mean ± standard deviation (SD) or ± Standard Error of the Mean (SEM). Statistical significance was determined using Student's *t*‐test or one‐way ANOVA with post hoc tests, where appropriate, using the graphpad prism software. Statistical significance was set at *P* < 0.05.

## 3 Results

### 3.1 EpCAM expression is heterogeneous across lung cancer cohorts

Although EpCAM is generally overexpressed in numerous epithelial‐derived tumors [33, 34, 35], its expression in lung cancer is variable, making its clinical interpretation challenging [26]. To evaluate EpCAM expression in lung cancer, we analyzed its expression in multiple lung cancer patient cohorts and cell line datasets. To categorize the tumor types as either epithelial or mesenchymal, we included a 76‐gene epithelial and mesenchymal signature panel previously established for NSCLC [36]. In the two tumor‐normal comparison cohorts [37, 38], EpCAM was consistently upregulated in tumors relative to normal tissues (Fig. 1A). Notably, lung adenocarcinomas and squamous cell carcinomas exhibited wide interquartile ranges for EpCAM expression (Fig. 1A). Comparative analysis across oncogenic mutations of ALK, KRAS, EGFR, and five lung cancer cohorts [39, 40, 41, 42, 43] revealed substantial heterogeneity in EpCAM expression (***P < 0.01, **P > 0.05, *P = ns) (Fig. 1B). Additional cohorts stratified by tumor stage [44], oncogenic mutations [45] and cancer type [46] supported this heterogeneity (Fig. 1C). Although some data sets were not statistically significant (*P > 0.5), there was a consistent trend of EpCAM upregulation in tumors compared to the matched normal tissue. In addition, approximately 20–30% of the tumor cell lines demonstrated either low or absent EpCAM expression (Fig. 1D). Cell lines lacking EpCAM displayed elevated levels of EGFR and AXL (Fig. 1E).

**Fig. 1:**
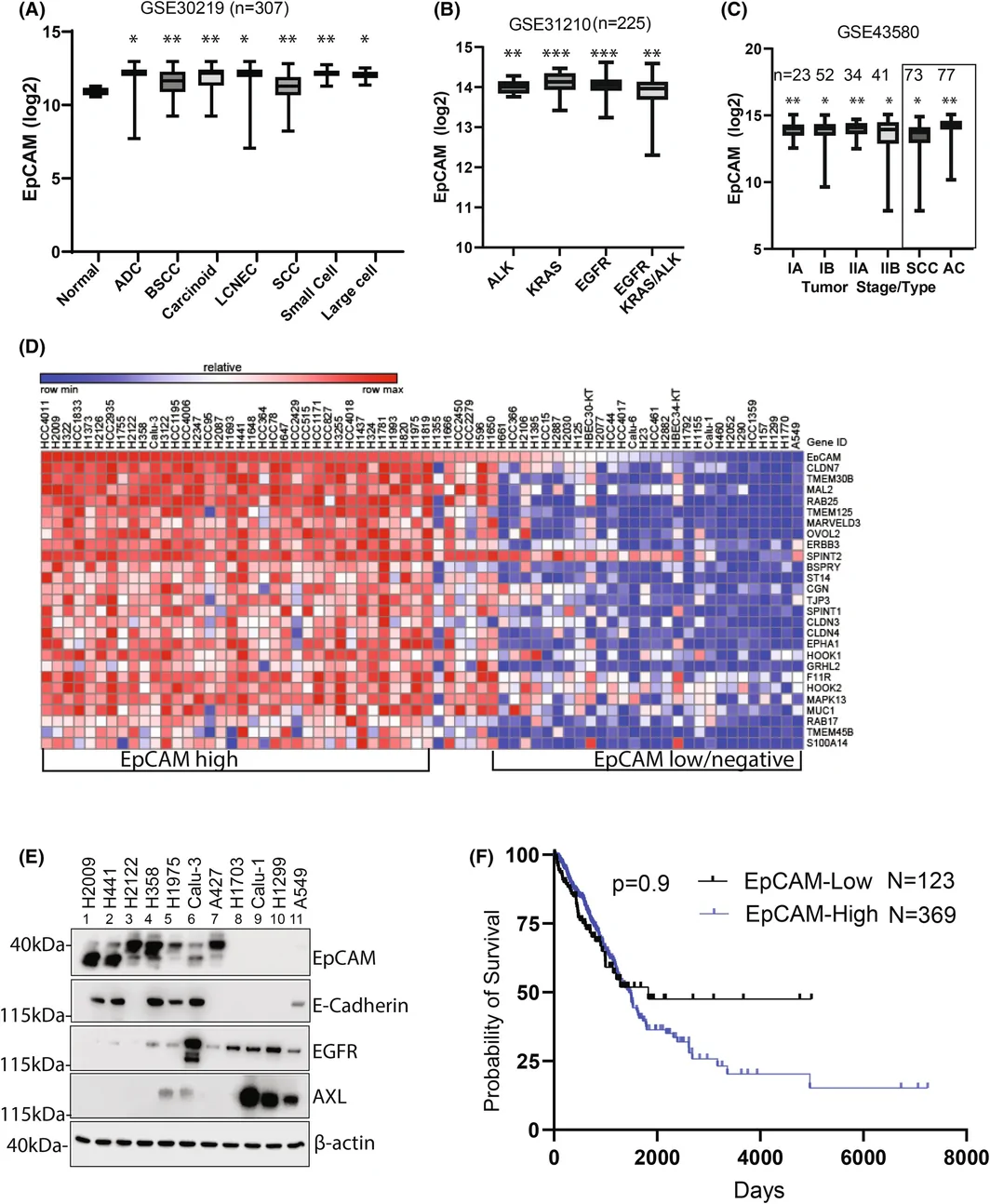
Heterogeneity of EpCAM expression in non‐small cell lung cancer (NSCLC). (A) EpCAM expression levels in normal lung tissue and various NSCLC histological subtypes from the GSE30219 cohort (*n* = 307). Expression values are shown as log2‐transformed normalized counts. Subtypes include Adenocarcinoma (ADC), Basaloid (BSCC), Carcinoid, Large Cell Neuroendocrine Carcinoma (LCNEC), Squamous Cell Carcinoma (SCC), Small Cell, and Large Cell lung cancers. (B) EpCAM expression variability across NSCLC tumors harboring common oncogenic drivers (ALK fusion, KRAS, and EGFR mutations) using the GSE31210 cohort (*n* = 225). (C) Distribution of EpCAM expression across lung cancer subtypes from the GSE43580 cohort (*n* = 300). (D) EpCAM expression distribution in NSCLC cell lines from the GSE32989 dataset, heat plot categorized into “EpCAM‐high” and “EpCAM‐low” groups based on log_2_ expression. (E) EpCAM protein expression in common lung cancer cell lines, correlated with epithelial (E‐cadherin, EpCAM positive) and mesenchymal (AXL‐positive) markers. Data represent three independent biological replicates. (F) Kaplan–Meier overall survival analysis of patients from the TCGA‐LUAD dataset (*n* = 492). Patients are stratified by the top quartile (75th percentile) versus the lower three quartiles of EpCAM expression. Statistical Analysis and Plotting: In panels (A–C), differences in log_2_‐transformed EPCAM expression were evaluated using a one‐way ANOVA followed by Tukey's post‐hoc test; significant differences (*P* < 0.05) are indicated. Box plots represent the median, interquartile range (IQR), and Tukey whiskers (1.5× IQR); outliers (values > 1.5× IQR) are plotted as individual points. In panel (F), Survival analysis from the TCGA LUAD dataset (*n* = 492) shows that individuals in the top quartile (75th percentile) for EpCAM expression have significantly reduced overall survival compared to those with low EpCAM expression. Differences between groups were assessed using the Mantel‐Cox (Log‐rank) test (*P* = 0.9). Univariate Cox proportional hazards regression shows a hazard ratio (HR) of 1.26 (95% CI: 0.89–1.78).

To investigate EpCAM expression relative to patient survival, we analyzed the TCGA adenocarcinoma dataset of approximately 500 patients. A substantial majority (75%) of patients exhibited EpCAM upregulation with poorer survival outcomes (*P* = 0.9) (Fig. 1F). For EpCAM expression, univariate Kaplan–Meier analysis revealed a median survival of 1454 days for patients with high EpCAM expression compared to 1830 days for those with low EpCAM expression. However, the difference in overall survival between these groups was not statistically significant (Mantel‐Cox test, *P* = 0.9). When low EpCAM expression was set as the reference group, the hazard ratio for high EpCAM expression was 1.26 (95% CI: 0.89–1.78), indicating a trend toward poorer survival in the high EpCAM group. Further analysis showed an inverse correlation between EpCAM expression levels (low, moderate, and high) and survival, where increased EpCAM expression correlated with decreased survival (Fig. S1A,B). The data also revealed a negative correlation between EpCAM and the mesenchymal marker Vimentin (Fig. S1B–D). The dynamic expression of EpCAM was also observed in cell lines with different cancer origins (Fig. S1E). Overall, our data indicate that EpCAM expression in NSCLC is dynamic, and its overexpression is linked to poor survival.

### 3.2 EpCAM modulates oncogenic signaling pathways and regulates AXL expression

Based on the EpCAM crystal structure (4MZV), molecular modeling, and previous reports [16, 25, 47], a schematic representation of EpCAM is presented in Fig. 2A. It contains ND (N‐terminal), thyroglobulin‐type 1 domain (TY), and CD (C‐terminal domain). To dissect the functional role of EpCAM, we engineered a series of EpCAM deletion mutants designed to mimic cleavage events mediated by ADAM17 and PSEN2, enzymes known to generate distinct EpCAM fragments with varying signaling capabilities [16]. These constructs were tagged C‐terminally with FLAG to facilitate protein detection. Specifically, we generated EpCAM‐314F: full‐length EpCAM (aa 1–314); EpCAM‐288F, which lacks the 26‐amino acid cytosolic domain; EpCAM‐265F, representing the N‐terminal soluble ectodomain; EpCAM‐140WF (aa 1–140), containing only the TY‐1 domain; and its proteolytic‐resistant mutant EpCAM‐140MF (R81I) (Fig. 2B). For this study, we utilized mixed populations after antibiotic selection to observe general rather than clonal phenotypic changes. Protein expression of these constructs was confirmed by retroviral transduction, followed by immunoblotting of A549 cells. Whole‐cell lysates were analyzed for EpCAM‐314F and EpCAM‐288F, whereas conditioned media was used for EpCAM‐265F and EpCAM‐140F (Fig. 2C). This process successfully confirmed the secretion of soluble EpCAM fragments. Stable expression of these EpCAM variants in A549 cells was achieved through retroviral transduction followed by antibiotic selection. Functional assays were conducted to evaluate the effect of EpCAM fragments on tumor cell behavior. In Matrigel invasion chambers, cells expressing soluble N‐terminal EpCAM‐265F exhibited a pronounced increase in invasion and migration compared to control GFP cells (Fig. 2D,E). An endpoint analysis revealed that EpCAM‐314 and EpCAM‐265 cells displayed significantly higher proliferation rates compared to control GFP cells, with increases of 13% (*P* = 0.03) and 28% (*P* = 0.001), respectively. Furthermore, the proliferation of both EpCAM‐265 and EpCAM‐314 consistently exceeded that of the GFP control and EpCAM‐288F cells (Fig. 2F). Additionally, in the wound‐healing assays, EpCAM‐265F cells demonstrated accelerated wound closure (Fig. 2G). Remarkably, under nutrient‐restricted conditions of 2% serum, all cell clones, except those expressing EpCAM‐265F, failed to survive, indicating that soluble EpCAM confers a significant survival advantage under stressful conditions (Fig. 2G, bottom panel). To note, sEpCAM (soluble EpCAM or EpCAM‐265F) has been detected in the ascitic fluid and serum of patients with epithelial malignancies [48, 49].

**Fig. 2:**
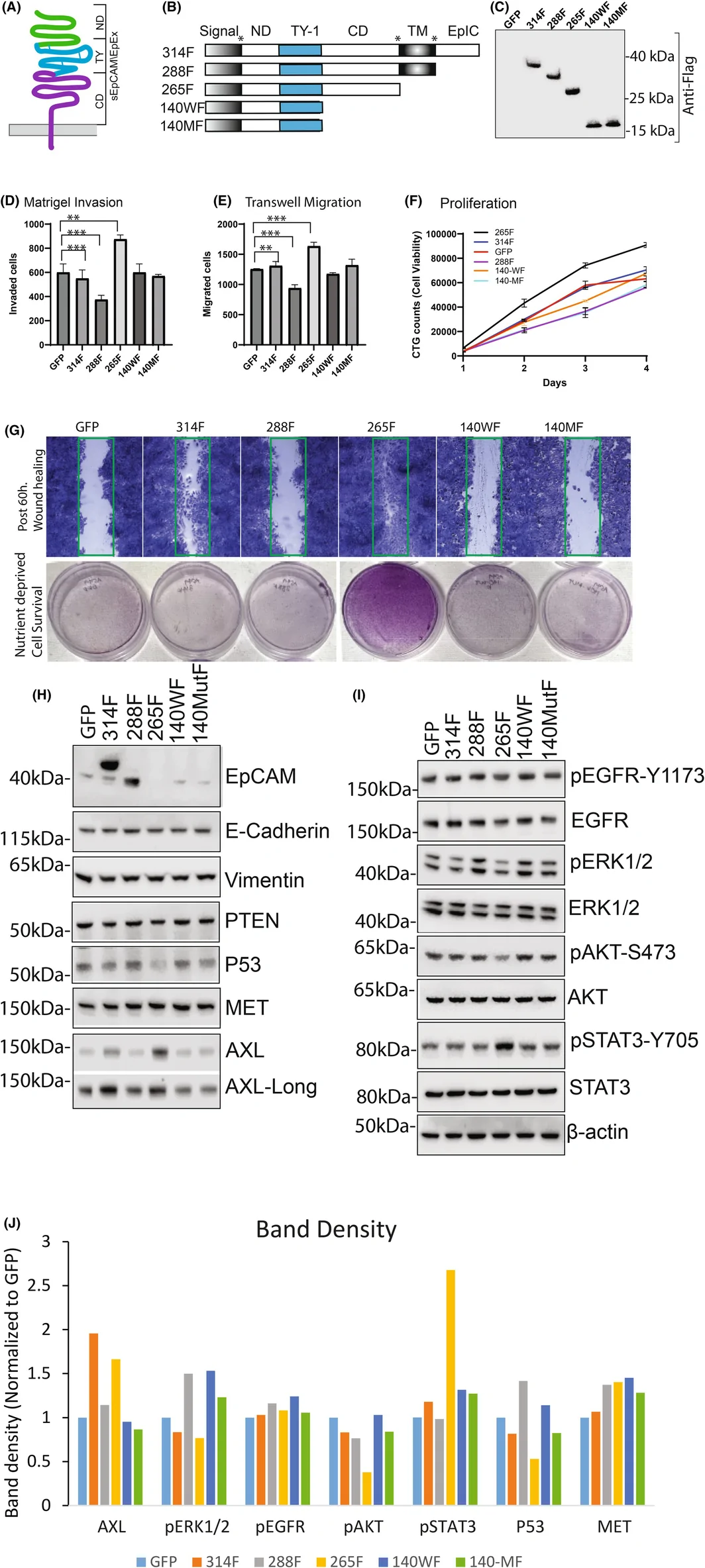
Impact of soluble EpCAM (EpCAM‐265F) on the phenotype and signaling. (A, B) Schematic representations of the full‐length EpCAM (EpCAM‐314F) protein and engineered deletion mutants, including EpCAM‐288F (cytosolic domain deletion), EpCAM‐265F (soluble N‐terminal ectodomain), and EpCAM‐140F (TY‐1 domain only). EpCAM‐140MF denotes the R81I mutant. ADAM17 and PSEN2 cleavage sites are denoted. All constructs are C‐terminally FLAG‐tagged. (C) Western blot analysis of whole‐cell lysates (for membrane‐bound variants) and concentrated conditioned media (for soluble ectodomain variants) from stable A549 cell lines. Proteins were detected via anti‐FLAG antibody. The representative blot shown is from three independent experiments. (D) Matrigel invasion assay quantifying the migratory capacity of A549 cells expressing EpCAM‐265F versus GFP‐control cells. Data are presented as relative invasion index normalized to the GFP control. Functional assessment of EpCAM‐265F on (E) cell migration (via transwell assay) and (F) proliferation CellTiter‐Glo assay. (G) Wound‐healing scratch assay (top) quantifying the percentage of wound closure over time, and (bottom) cell viability assessment under nutrient‐deprivation stress (72 h) in A549 cells expressing EpCAM‐265F versus control. (H, I) Western blot analysis of signaling molecules in A549 cells expressing the indicated EpCAM variants. (H, I) Representative immunoblots for E‐cadherin, Vimentin, c‐Met, AXL, p53, total/phosphorylated EGFR, ERK, AKT, and STAT3. GAPDH or β‐Actin was used as an internal loading control. (J) Densitometric quantification of protein expression/phosphorylation levels relative to loading controls. All experiments were performed in three independent biological replicates. Data are presented as mean ± SD. In (D–F), significance was determined by a two‐way ANOVA (mixed‐effects model) followed by a paired *t*‐test for specific group comparisons (*P < 0.1, **P < 0.05, ***P < 0.001).

To examine the impact of EpCAM on signaling, we analyzed A549 stable cell lines expressing various EpCAM deletion constructs (see Fig. 2A–C). Whole‐cell lysates from proliferating cells were subjected to immunoblotting to assess changes in key signaling molecules. As shown in Fig. 2H, the expression of E‐cadherin and Vimentin remained unchanged across all variants, indicating that the EMT status was not significantly affected. Similarly, the expression of c‐Met, a drug resistance marker, marginally changed. Notably, p53 expression was decreased in both full‐length EpCAM and EpCAM‐265F expressing cells. The most striking change was observed in AXL expression, which was upregulated in EpCAM‐314F and EpCAM‐265F expressing cells. Analysis of the canonical growth factor pathways revealed that EGFR expression and activation were not significantly altered. However, both ERK and AKT phosphorylation were decreased in the EpCAM‐265 cells. Interestingly, we observed increased activation of STAT3 in the EpCAM‐265F lysate (Fig. 2I,J).

### 3.3 Tumor microenvironment induces EpCAM expression, promoting AXL expression, modulates ERK, NF‐kB, c‐Myc, and STAT3 signaling

We frequently observed elevated EpCAM expression in xenografted tumor cells compared to 2D cultured cells. Lung cancer cell lines A549, H1299, and H1975 exhibited minimal baseline EpCAM expression under 2D conditions. These cells, either wild‐type or expressing GFP‐puromycin, were implanted subcutaneously into immunocompromised mice and subsequently recovered after 4 weeks (see Methods). Quantitative gene expression from tumor‐derived and 2D cells was obtained via qRT‐PCR. Fold changes were calculated using the ddCT method (ddCT tumor‐derived RNA/ddCT parental 2D RNA). We identified significant upregulation of EpCAM transcripts in A549 cells (3‐fold), H1299 (5‐fold), and H1975 (2.5‐fold) in tumor‐derived cells compared to 2D culture (*P* < 0.05). We did not observe a significant increase in expression of epithelial and mesenchymal markers, including E‐cadherin, MAPK13, Vimentin, and AXL (Fig. 3A). Next, we generated tumor‐derived A549 cells expressing control GFP and EpCAM variants 314F, 288F, and 265F. Immunoblotting verified increased EpCAM expression in tumor‐derived GFP cells when compared to GFP‐2D cells. However, AXL expression decreased in GFP‐Tu cells relative to that in GFP‐2D cultured cells (Fig. 3B). Tumor‐derived A549‐314F (full‐length EpCAM) cells showed prominent cleaved or glycosylated forms. Surprisingly, the EpCAM‐265F construct expressing sEpCAM induced endogenous EpCAM expression along with increased AXL expression. EpCAM‐288F cell lysate did not show changes to endogenous EpCAM or AXL expression. Since EpCAM‐140F did not exhibit any phenotypic changes or impact on intracellular signaling (see Fig. 2D–G), we focused our investigation on the more relevant forms of EpCAM‐314F and EpCAM‐265F. For all subsequent experiments, tumor‐derived cells were utilized as a more relevant study model.

**Fig. 3:**
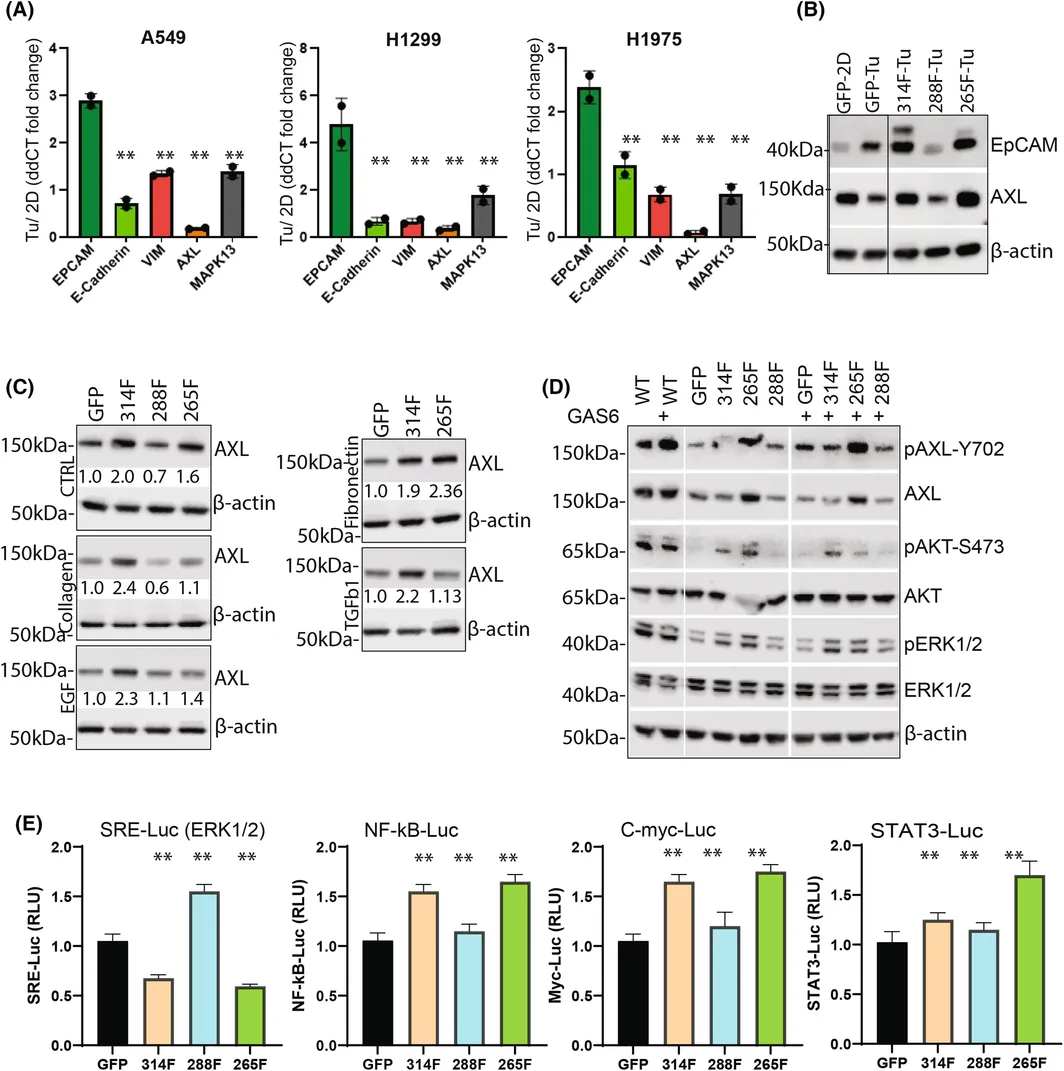
Modulation of EpCAM expression and signaling by the tumor microenvironment (TME). (A) Quantitative RT‐PCR analysis of EPCAM, E‐cadherin, VIM (Vimentin), AXL, and MAPK13 mRNA levels in A549, H1299, and H1975 cell lines. Data are presented as fold‐change relative to 2D‐cultured control cells, calculated using the $2^{-\Delta \Delta C_{t}}$ method. (B) Western blot analysis of A549 cells expressing EpCAM variants, demonstrating regulation of AXL and endogenous EpCAM protein levels. (C) Effect of TME‐mimetic stimuli on AXL protein expression. A549 cells expressing EpCAM‐314F or EpCAM‐265F were cultured in the presence of collagen, fibronectin, EGF, or TGF‐β. (D) Assessment of EpCAM‐mediated AXL signaling. A549 cells expressing GFP, EpCAM‐314F, or EpCAM‐265F were stimulated with GAS6 (the AXL ligand). Phosphorylation of AXL, AKT, and ERK1/2 was evaluated by western blot. (E) Transcriptional activity of pro‐tumorigenic pathways. Luciferase reporter assays were performed in A549 cells to measure the activity of NF‐κB, Myc, STAT3, and ERK/SRE signaling in the presence of EpCAM‐265F compared to the GFP control. Panel B–D, representative blot shown is from three independent experiments. Statistical Analysis: Data are representative of three independent biological replicates. Bar graphs are presented as mean ± SD (A) or mean ± SEM (E). Statistical significance was assessed using an unpaired, two‐tailed Student's *t*‐test comparing experimental groups to the respective controls (**P < 0.05).

Given that EpCAM expression induced AXL upregulation, we next investigated whether AXL expression could be further modulated by components of the tumor microenvironment. Tumor‐derived A549 cells expressing various EpCAM constructs were cultured for 24 h on collagen‐ or fibronectin‐coated plates or were treated with EGF or TGF‐β. Immunoblot analysis of the cell lysates (Fig. 3C) revealed that EpCAM‐314F consistently induced AXL upregulation across all tested microenvironmental stimuli. However, cells expressing EpCAM‐265F only showed increased AXL expression when cultured on fibronectin. These results highlight the context‐dependent interaction between EpCAM signaling and the extracellular matrix components.

AXL activation, crucial for various cellular processes, can be triggered by homodimerization, heterodimerization with other TAM receptors, or binding to its primary ligand, growth arrest‐specific‐6 (GAS6). To investigate AXL activation in EpCAM‐expressing cells, we serum‐starved cells overnight (2% serum) and then treated them with recombinant GAS6. As expected, GAS6 treatment induced AXL phosphorylation across all A549 cell lines harboring EpCAM deletion mutants. Notably, in EpCAM‐265F‐expressing cells, GAS6 stimulation further enhanced AXL phosphorylation, demonstrating that a soluble form of EpCAM (EpCAM‐265F) can contribute to the functional activation of the AXL signaling pathway. Intriguingly, both tumor‐derived EpCAM‐314F and EpCAM‐265F cells consistently displayed elevated levels of phosphorylated AKT and ERK, irrespective of GAS6 treatment (Fig. 3D). To further validate these findings, we performed a luciferase‐based reporter assay to assess signaling pathway activation. Tumor‐derived A549 cells expressing EpCAM constructs were transiently transfected with firefly luciferase reporters driven by response elements specific for ERK (SRE‐Luc), NF‐κB, Myc, and STAT3, along with a TK‐Renilla luciferase construct for internal normalization. Dual‐luciferase assays revealed that EpCAM‐265F cells increased the transcriptional activity of NF‐κB, Myc, and STAT3 (Fig. 3E).

### 3.4 EpCAM and sEpCAM expression exhibit characteristics of a drug‐resistant phenotype

Initial characterization of these tumor‐derived cell lines revealed elevated phosphorylation of key signaling proteins, including AXL, AKT, and ERK (Fig. 3D). The activation of these pathways is frequently associated with increased cell survival, proliferation, and drug resistance. To assess the drug sensitivity profile of tumor‐derived A549 cells, the cells were treated for 48 h with a panel of pathway‐specific inhibitors at their reported IC50 concentrations (Table S1 and Fig. 4A). These inhibitors target diverse oncogenic signaling pathways, including PLK1, proteasome/NF‐κB, EGFR, c‐MET, ATPase, Src, mTOR, MAPKs, HSP90, ROCK, and chemotherapeutic drugs. CellTiter‐Glo‐based viability assays for 33 drugs revealed modest and variable effects across EpCAM constructs. A549 cells expressing GFP and EpCAM‐288F were sensitive to 17‐DMAG, Crizotinib, BI‐2536, Bortezomib, and Gemcitabine. However, this sensitivity decreased by approximately 50% in EpCAM‐314F and sEpCAM‐265F cells (Fig. 4A). These results were further validated in 12‐well cultures after 72 h of drug treatment. Cell survival was assessed by crystal violet staining, which consistently validated that EpCAM‐314F and EpCAM‐265F conferred a significant survival advantage when exposed to these therapeutic agents (Fig. 4B). Titration experiments performed across a range of 0.1 nm to 1000 nm of 17‐DMAG, Gemcitabine, and Crizotinib further substantiated these findings. We consistently observed increased resistance to multiple drugs in cells expressing EpCAM‐314F or EpCAM‐265F compared with GFP controls (Fig. 4C–E and Fig. S2A–H). The enhanced survival rate was particularly remarkable in the presence of crizotinib. Quantitative analysis of crizotinib sensitivity further underscored the profound impact of EpCAM expression. The IC50 for Crizotinib in A549‐GFP cells was determined to be approximately 7.5 nm. In stark contrast, the IC50 for Crizotinib was dramatically elevated to approximately 1000 nm in both A549‐EpCAM‐314F and A549‐sEpCAM‐265F cell lines (Fig. 4E). This represents a 130‐fold increase in crizotinib resistance mediated by the expression of full‐length or sEpCAM, providing compelling evidence of their involvement in drug resistance. Interestingly, the A549‐EpCAM‐288F cell line, which expresses EpCAM lacking its intracellular domain, exhibited drug sensitivity similar to that of A549‐GFP control cells across most tested agents (Fig. 4A,B). Results also suggest that crizotinib reduced cell growth (Fig. 4B) rather than cell death.

**Fig. 4:**
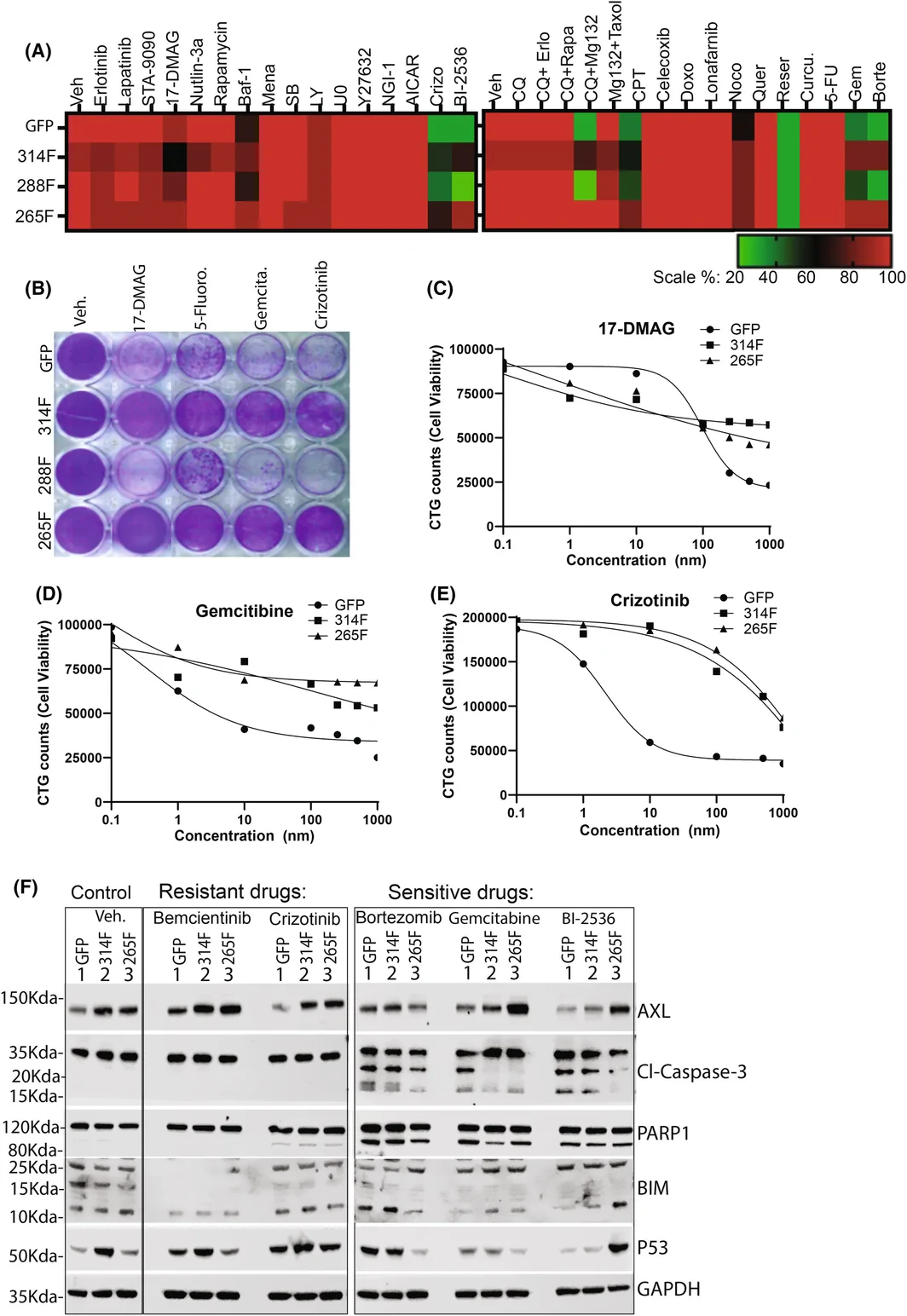
EpCAM and sEpCAM expression confer drug resistance in A549 cells. (A) Heat map illustrating the differential viability of A549‐GFP (control), A549‐EpCAM (314F), and A549‐sEpCAM (265F) cells after a 48‐h treatment with a panel of pathway‐specific inhibitors at their respective IC50 concentrations. Green indicates reduced cell viability, while red indicates increased viability compared to controls. (B) Representative crystal violet staining of cell colonies following treatment. (C–E) Quantitative analysis of cell survival for A549‐GFP, A549‐EpCAM‐314F, A549‐sEpCAM‐265F, and A549‐EpCAM‐288F cells treated with 17‐DMAG (C), Gemcitabine (D), and Crizotinib (E) for 72 h. The shift in Crizotinib IC50 values in EpCAM‐ and sEpCAM‐expressing cells is highlighted. Data are presented as mean ± SEM from three independent biological replicates (*n* = 3), each performed in technical triplicate. (F) Immunoblot analysis of AXL, cleaved Caspase‐3, PARP1, BIM, and P53 in A549‐GFP, A549‐EpCAM‐314F, and A549‐sEpCAM‐265F cells following drug treatment, demonstrating differential apoptotic induction. GAPDH was used as a loading control. Representative blots from three independent experiments are shown.

Next, we examined cell viability following Crizotinib treatment. While stable EpCAM knockdown alone (shEpCAM) significantly sensitized A549‐265F cells to Crizotinib (Fig. S3B), we hypothesized that this increased sensitivity was mediated through AXL. To test this, we transiently reintroduced AXL into the EpCAM‐depleted A549‐265F cells using AXL expression plasmid. Immunoblotting demonstrated successful re‐expression of AXL in these cells (Fig. S3A). Strikingly, re‐expression of AXL in EpCAM knockdown cells significantly restored Crizotinib resistance, reversing the increased sensitivity observed with EpCAM ablation alone (Fig. S3B). These results demonstrate that the Crizotinib sensitivity incurred by EpCAM loss is indeed dependent on the presence of AXL, further underscoring the functional link between EpCAM and AXL in drug resistance.

We analyzed key apoptotic markers to determine whether drug‐mediated apoptosis was induced in EpCAM‐expressing A549 cells. Among the tested drugs (Bemcentinib, Bortezomib, Gemcitabine, Crizotinib, BI‐2536), Bortezomib, Gemcitabine, and BI‐2536 significantly induced cell death as evidenced by robust Caspase‐3 and PARP1 cleavage at two‐fold their respective IC50 values. Surprisingly, Bemcentinib, an AXL kinase inhibitor, failed to induce cell death as a monotherapy. Crizotinib‐induced marginal apoptosis (Fig. 4F). This observation, in conjunction with our findings that EpCAM expression stabilizes AXL, suggests that EpCAM mediates resistance to crizotinib‐induced apoptosis by preventing sufficient AXL downregulation or by maintaining pro‐survival AXL signaling. Conversely, our findings indicate that drug interventions such as Bortezomib, Gemcitabine, and BI‐2536 can effectively overcome EpCAM‐mediated survival and resistance, leading to the induction of apoptosis.

### 3.5 sEpCAM enhances AXL stability by modulating the ubiquitin‐proteasome pathway

Given that sEpCAM/EpCAM‐265F promotes multidrug resistance and is susceptible to Bortezomib or BI‐2536 (Fig. 4F), we investigated whether these drugs altered AXL protein expression. Tumor‐derived A549 cell variants were treated for 48 h with a panel of inhibitors at reported IC50 doses. These inhibitors included BI‐2536 (PLK1 inhibitor), Bortezomib (proteasome/NF‐κB inhibitor), Afatinib (EGFR inhibitor), Crizotinib (c‐MET inhibitor), Dasatinib (Src family kinase inhibitor), Rapamycin (mTOR inhibitor), Trametinib (MEK inhibitor), and Y‐27632 (ROCK inhibitor). Immunoblotting revealed that, compared to other inhibitors which had modest or variable effects on AXL expression, BI‐2536 and Bortezomib reduced AXL expression and ERK activation in EpCAM‐265F‐expressing cells. Quantification of immunoblot band density confirmed a prominent decrease in AXL levels, with BI‐2536 and Bortezomib treatment resulting in a three‐ and five‐fold reduction, respectively (Fig. 5A,B). Next, tumor‐derived A549 cells (expressing GFP, 314F, or 265F) were treated with 100‐nm Bortezomib for 2, 5, and 10 h. Immunoblot analysis of cell lysates revealed a consistent time‐dependent reduction in AXL expression across all cell lines, regardless of EpCAM expression (Fig. 5C,D). The band density data indicated that AXL exhibited significantly higher baseline stability and resistance to degradation in the presence of EpCAM‐314F and particularly sEpCAM‐265F compared to GFP control cells.

**Fig. 5:**
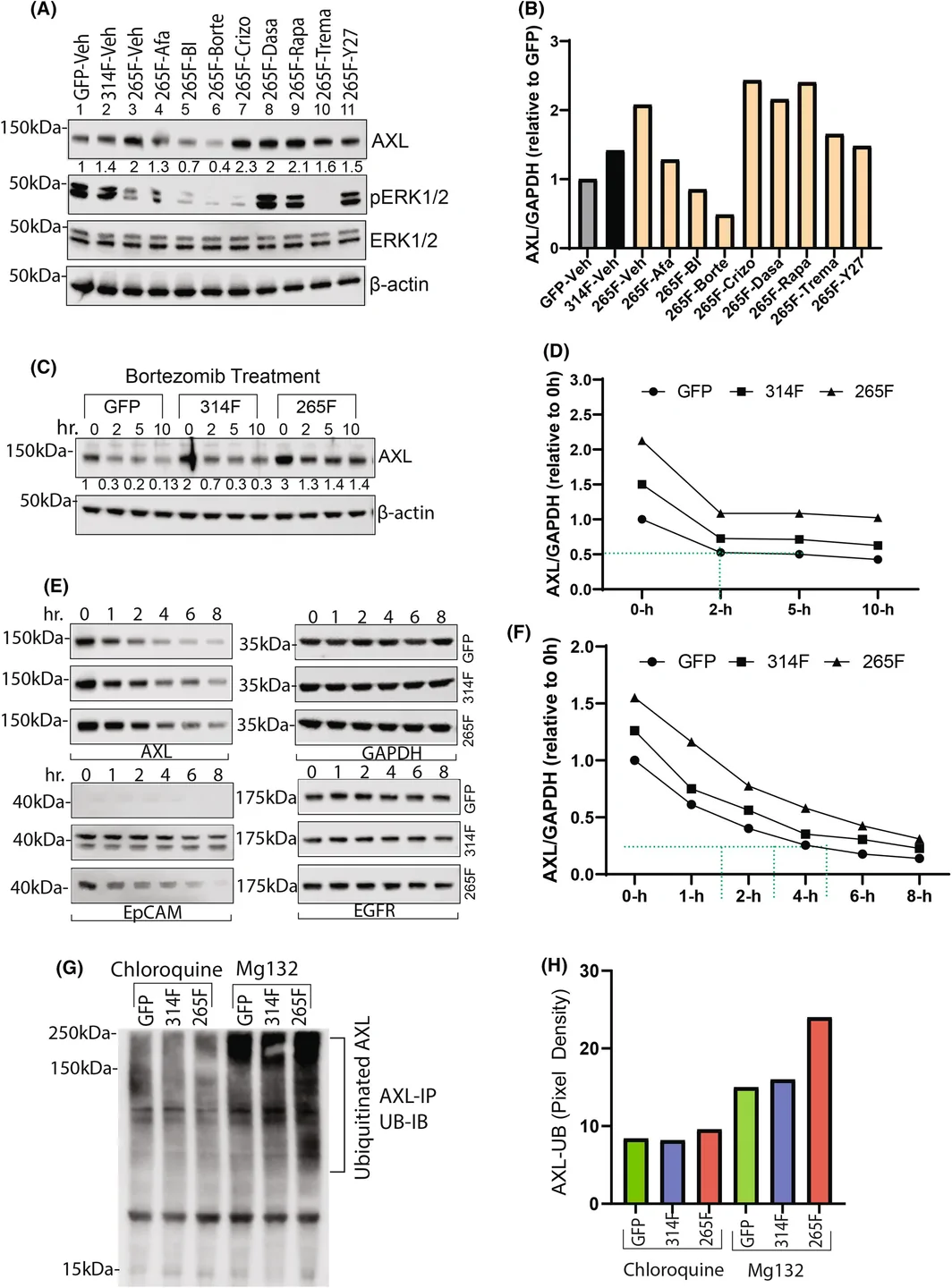
sEpCAM stabilizes AXL by hindering its proteasomal degradation. (A) Immunoblot analysis and (B) corresponding densitometric quantification of AXL protein levels in A549‐GFP, A549‐EpCAM‐314F, and A549‐sEpCAM‐265F cells treated with various pathway‐specific inhibitors at IC50 concentrations for 48 h. Note that BI‐2536 and Bortezomib treatment significantly reduce AXL expression in sEpCAM‐expressing cells. (C) Immunoblot analysis and (D) quantitative analysis of AXL protein levels over a 10‐h time course (2, 5, 10 h) following Bortezomib treatment. (E) Immunoblot analysis and (F) densitometric quantification of AXL levels following a cycloheximide (CHX) chase assay (0–10 h). AXL protein half‐life is progressively extended from A549‐GFP (~ 1.5 h) to A549‐EpCAM‐314F (~ 2.5 h) to A549‐sEpCAM‐265F (~ 5 h). (G) Immunoblot analysis of immunoprecipitated (IP) AXL and its ubiquitinated forms following treatment with MG132 or Chloroquine. (H) Densitometric quantification of ubiquitinated AXL levels. AXL antibody was used for immunoprecipitation of the lysates and blotted for Ubiquitin. Representative blots and results from three independent experiments are shown. GAPDH or β‐Actin was used as a loading control for all immunoblot analyses.

To investigate the degradation kinetics and stability of AXL, the half‐life of AXL was determined in A549 cells expressing GFP, EpCAM‐314F, or EpCAM‐265F. Cells were subjected to a cycloheximide (CHX) chase assay, in which protein synthesis was inhibited for 0, 1, 2, 5, 8, and 10 h. Subsequent immunoblotting and band density analysis of cell lysates revealed a baseline AXL half‐life of approximately 1.5 h in A549‐GFP control cells. The expression of EpCAM‐314F moderately increased AXL stability, extending its half‐life to 2.5 h. Notably, in cells expressing sEpCAM (265F), the half‐life of AXL was significantly prolonged to 5 h, indicating that sEpCAM strongly enhances AXL stability (Fig. 5E,F). This is in agreement with a previous report that in Gefitinib‐resistant NSCLC cells, the half‐life of the AXL protein was longer than 1.8 h in sensitive cells [50].

To further dissect EpCAM‐mediated proteasomal or lysosomal AXL stabilization across these cell lines, A549‐GFP, 314F, and 265F cells were treated with either the proteasome inhibitor MG132 or the lysosomal inhibitor chloroquine. Following treatment, the cell lysate was i*mmunoprecipitated* using an AXL antibody, followed by immunoblotting for ubiquitin to assess AXL ubiquitination. Immunoblot and band density analyses revealed significant accumulation of AXL and an increase in its ubiquitinated forms following MG132 treatment in EpCAM‐314F and EpCAM‐265F compared to GFP cells (Fig. 5G,H). This strongly suggests that primary degradation of AXL occurs via the ubiquitin‐proteasome system. This effect was particularly pronounced in EpCAM‐265F cells, consistent with the extended AXL half‐life shown previously (Fig. 5E). Conversely, chloroquine treatment resulted in only a modest increase in total AXL levels, indicating a minor, if any, contribution from the autophagy‐lysosome pathway.

### 3.6 EpCAM and AXL ablation sensitize tumor cells to drugs

As EpCAM upregulation stabilized AXL, which drives multidrug resistance, we used shRNA to ablate AXL and EpCAM. Tumor‐derived A549 expressing EpCAM‐314F and EpCAM 265F cell lines were transduced with previously validated shRNAs targeting either AXL or EpCAM [24, 51]. Immunoblotting of the lysates showed that lentiviral shRNA ablated more than 90% and 80% of the EpCAM and AXL proteins, respectively (Fig. 6A). Depletion of AXL and EpCAM had significant effects on BCL‐2 and Snail. BCL‐2 expression was significantly decreased in both AXL and EpCAM shRNA groups. Furthermore, Snail, a well‐established EMT driver, showed a 20–40% reduction with AXL shRNA and a 30–40% reduction with EpCAM shRNA, consistently observed across both EpCAM‐positive cell lines. To assess if AXL overexpression could modulate drug sensitivity in EpCAM‐ablated cells, we transiently transfected AXL into EpCAM shRNA cells. This resulted in increased cell viability in response to Crizotinib treatment (Fig. S3A,B). Next, we conducted two independent experiments to assess the functional consequences of these knockdowns on cell proliferation and drug sensitivity. For cell proliferation, 1 × 10^5^ cells were seeded at 15% confluence in 96‐well plates for 72 h, and viability was quantified using an ATP‐based CellTiter‐Glo assay. AXL shRNA suppressed cell growth by 40–60% in both EpCAM‐314F and EpCAM‐265F stable cell lines (Fig. 6B). In contrast, EpCAM shRNA demonstrated a more potent inhibitory effect, reducing cell growth by 60–75% in these respective cell populations. To evaluate the drug sensitivity of these knockdown cells, cells were plated to 50% density in a 12‐well plate and treated with Gemcitabine or Crizotinib for 48 h. Crystal violet staining revealed that cells with EpCAM knockdown were more sensitive to both Gemcitabine and Crizotinib than cells with AXL knockdown (Fig. 6C).

**Fig. 6:**
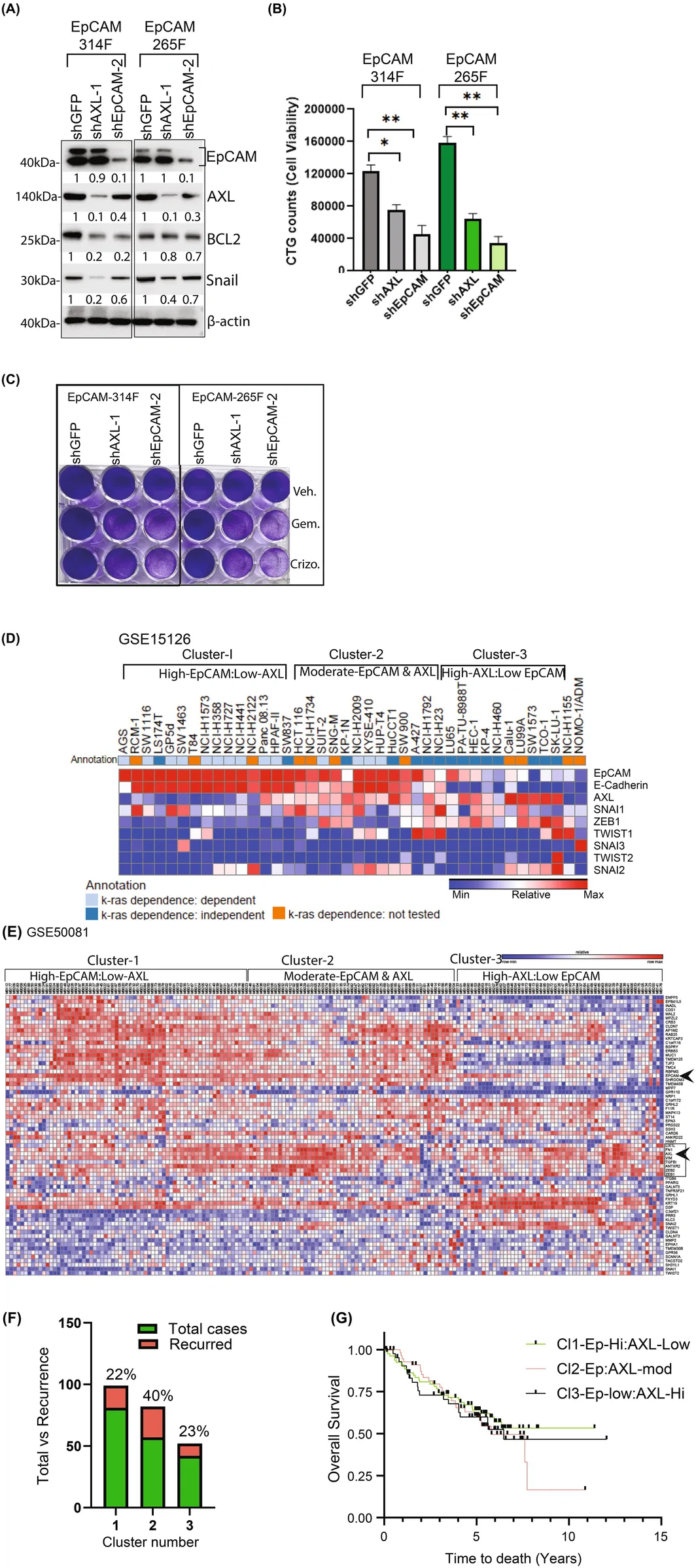
shRNA‐mediated knockdown of EpCAM or AXL sensitizes cancer cells to therapeutic agents and defines a clinical signature. (A) Immunoblot analysis confirming efficient shRNA‐mediated knockdown of AXL and EpCAM in A549‐EpCAM‐314F and A549‐sEpCAM‐265F cells. Modulation of Snail and BCL‐2 expression levels was also assessed. β‐Actin was used as the loading control. (B) CellTiter‐Glo assay showing cell growth inhibition in A549‐EpCAM‐314F and A549‐sEpCAM‐265F cells following knockdown of AXL or EpCAM. Data are presented as mean ± SD of three independent biological replicates (*n* = 3), each performed in technical triplicate. Statistical significance was determined by Student's *t*‐test (*P < 0.05, **P < 0.01). Results shown in panels A and B are representative of three independent experiments. (C) Representative crystal violet staining demonstrating increased sensitivity to Gemcitabine and Crizotinib in EpCAM‐knockdown cells compared to AXL‐knockdown and control cells. (D, E) Bioinformatics analysis of GEO datasets (GSE15126 and GSE50081) using the NSCLC 76‐gene EMT signature. Hierarchical cluster analysis segregates datasets from cell lines or patient samples into distinct groups, identifying a significant co‐expression signature for AXL and EpCAM. (F) Recurrence rates in TCGA lung adenocarcinoma (LUAD) patient cohorts based on the identified clusters; the AXL/EpCAM co‐expression group exhibits higher recurrence. (G) Kaplan–Meier overall survival analysis of patients stratified by AXL and EpCAM expression levels. Survival significance was calculated using the Mantel–Cox log‐rank test (*P* = 0.7). Representative results for panels A–C are shown from three independent biological replicates.

### 3.7 Identifying potential therapeutic vulnerable patient groups to target EpCAM‐AXL axis

Next, we sought to identify the NSCLC patient subset that expressed AXL and EpCAM. Interestingly, despite the overall inverse correlation between AXL and EpCAM expression (*n* = 166, *r* = −0.25, *P* < 0.001; Fig. S1C,D). To assess the prognostic value of this co‐expression, we analyzed recurrence and overall survival using the GEO dataset (GSE50081) with the NSCLC EMT signature [36, 40]. Hierarchical clustering resulted in the segregation of cell line or patient data into three clusters, revealing distinct molecular profiles (Fig. 6D,E). Cluster 1 displays a robust epithelial signature, marked by strong expression of EpCAM, E‐Cadherin, CLDN7, RAB25, and MUC1. Conversely, Cluster 3 shows a strong mesenchymal profile, evidenced by the enrichment of Vimentin, LIX1L, FN1, AXL, ZEB1/2, and TGFβ1. Cluster 2 appears to represent an intermediate state, co‐expressing these mesenchymal genes, including SNAI1, SNAI2, and TWIST1, while still retaining moderate EpCAM levels. Notably, Cluster 2, defined by concomitant AXL and EpCAM expression, correlated with aggressive disease, manifesting as a 40% recurrence rate (Fig. 6F) and reduced overall survival (Fig. 6G) when compared to Cluster 1 or 2. To investigate the impact of AXL and EpCAM co‐expression, we categorized patients into three groups: ‘AXL Low, EpCAM High’, ‘AXL‐EpCAM Moderate’, and ‘AXL High, EpCAM Low’. Patients in the ‘AXL Low, EpCAM High’ group showed a median survival of 1501 days, with a hazard ratio of 1.2 (95% CI: 0.82–1.7). The ‘AXL‐EpCAM Moderate’ group had a median survival of 1621 days and a hazard ratio of 1.2 (95% CI: 0.89–1.8). The ‘AXL High, EpCAM Low’ group exhibited the shortest median survival at 1221 days, corresponding to a hazard ratio of 0.84 (95% CI: 0.59–1.2). Pairwise comparisons using the Mantel‐Cox test revealed no statistically significant differences in overall survival between the ‘AXL‐EpCAM Moderate’ and ‘AXL High, EpCAM Low’ groups (*P* = 0.4), nor between the ‘AXL Low, EpCAM High’ and ‘AXL High, EpCAM Low’ groups (*P* = 0.7).

To generate an EpCAM‐AXL‐driven gene signature capable of guiding therapy for a specific patient subgroup, we focused on cluster‐2 (Fig. 6D,E). Our initial analysis utilized a less noisy NSCLC cell line gene expression dataset (GSE4824). Close correlation analysis allowed us to identify gene sets that were significantly correlated (both positively and negatively) with AXL and EpCAM expression (Fig. S4A). We enriched this gene set/signature by combining it with a larger NSCLC dataset (Fig. S4B). Gene Ontology (GO) analysis of the 24‐gene EpCAM‐AXL signature showed significant enrichment in p53‐associated, integrin signaling, Rho GTPase, and altered immune response pathways (Fig. S4C). This analysis not only identifies p53, integrin, and Rho GTPase‐mediated signaling as its core components but also points to platelet involvement, which aids in binding and protecting tumor cells from immune surveillance [52, 53].

## 4 Discussion

Our study uncovers an EpCAM‐AXL axis that may drive drug resistance in lung cancer cells harboring oncogenic mutations, revealing a previously unrecognized role for EpCAM in stabilizing AXL. This mechanistic insight not only identifies a clinically aggressive patient subset but also highlights actionable vulnerabilities, offering new avenues for biomarker development and therapeutic intervention. In agreement with existing literature, our findings confirmed the significant heterogeneity of EpCAM expression across various lung cancer patient cohorts and cell lines [20, 26, 54, 55]. Non‐parametric analysis using the Kruskal–Wallis test indicated a statistically significant overall difference in EpCAM expression among all four oncogenic groups. Post‐hoc pairwise comparisons further revealed significant differences in EpCAM expression: between the ALK, EGFR, and KRAS groups (*P* = 0.0014), and between the EGFR and KRAS groups (*P* = 0.0005) (Fig. 1A–C). We demonstrated that this variability is not merely a static feature but a dynamic response that is heavily influenced by the tumor microenvironment. Specifically, the co‐downregulation of EpCAM and E‐cadherin in approximately 40% of lung cancer cell lines supports EMT‐mediated suppression of these markers. The inverse correlation between increased EpCAM expression and decreased survival in TCGA adenocarcinoma patients (Fig. 1F and Fig. S1A) and its negative correlation with Vimentin expression (Fig. S1B) highlights the complex prognostic role of EpCAM in NSCLC. Our data also highlighted that the tumor microenvironment profoundly reprograms EpCAM expression. Cell lines exhibiting low basal EpCAM showed substantial upregulation and concurrent downregulation of AXL when they were grown as subcutaneous tumors (Fig. 3A–D), implicating stromal cues, ECM components, and cytokines in epithelial reprogramming.

Our study further delineates the critical role of sEpCAM, generated by ADAM17‐mediated proteolytic shedding [16, 49], as an active signaling molecule. Consistent with its reported presence in malignant ascites and prognostic value [48], we demonstrated that sEpCAM significantly enhanced cancer cell migration, invasion, and proliferation (Fig. 2C–E). Interestingly, sEpCAM also promoted survival under nutrient‐deprived conditions (Fig. 2F). This establishes sEpCAM as a potent pro‐tumorigenic entity, corroborating its utility as a non‐invasive biomarker [48, 56]. In the present study, an RTK switch involving MET or EGFR signaling, a commonly recognized adaptive mechanism, was not detected (Fig. 2H,I).

In cancer cells, AXL expression is tightly regulated at multiple levels, including epigenetic alterations, transcriptional control by factors like HIF‐α, AP‐1, NF‐κB and YAP1/TEAD, CREB, SP1, and post‐transcriptional modulation by miRNAs [57]. Our previous results show that RAS‐transformed primary cells often activate AP‐1 and NF‐κB signaling [23, 24]. To our knowledge, this is the first study to reveal a crucial and previously unrecognized regulatory link between EpCAM and AXL. EpCAM consistently induced AXL upregulation in full‐length EpCAM and sEpCAM‐expressing cells (Fig. 2G) and markedly prolonged the AXL half‐life from ~ 1.5 to ~ 5 h (Figs 3F, 5E–G). This sustained AXL signaling, even without direct ligand stimulation, activates the PI3K/AKT and MAPK/ERK pathways. This occurs across diverse microenvironmental stimuli and when cells are cultured on extracellular matrices (Fig. 3E), indicating a cooperative signaling axis between EpCAM, AXL, and extracellular matrix components that drives tumor adaptation. Concurrently, EpCAM expression activated NF‐κB, Myc, and STAT3 (Fig. 3G–J), collectively reinforcing survival, immune evasion, and therapy‐resistant phenotypes. Our initial experimental design aimed to compare the effects of EpCAM and EpCAM‐265 in both 2D culture and *in vivo* tumor settings. Although not all EpCAM construct variants were assessed side‐by‐side in the same 2D experiment using tumor‐derived cells, we consistently employed GFP‐expressing cells as an internal control to account for microenvironmental influences. Notably, GFP‐expressing cells exhibited lower AXL expression in tumor‐derived settings relative to 2D culture, suggesting that the tumor microenvironment modulates baseline AXL levels. Importantly, however, the pattern of AXL upregulation observed in EpCAM‐314F and EpCAM‐265F cells remained consistent across both 2D culture and tumor‐derived conditions. This consistency indicates that, while the tumor microenvironment may broadly influence AXL expression levels, the specific upregulation driven by EpCAM‐314F and EpCAM‐265F persists regardless of context.

Our data reveal for the first time that the EpCAM‐AXL axis directly mediates functional drug resistance. Specifically, EpCAM or sEpCAM expressing cells demonstrated broad resistance to multiple targeted agents, including over a 130‐fold increase in crizotinib IC50 compared to controls (Fig. 4A–C,E). This robust resistance specifically required the cytosolic tail of EpCAM as the truncated form remained sensitive. Importantly, we identified a key actionable vulnerability; these resistant cells demonstrated increased sensitivity to PLK1 and proteasome inhibitors, both of which efficiently reduced AXL protein levels (Figs 4A, 5A,B). Knockdown studies further revealed that while AXL depletion improved crizotinib sensitivity, EpCAM knockdown produced greater sensitization to crizotinib and Gemcitabine (Fig. 6A–C), indicating that EpCAM exerts dominant control over resistance through AXL‐dependent and AXL‐independent mechanisms. This provides a compelling rationale for proteasome inhibition as a therapeutic strategy for EpCAM/AXL‐driven tumors. Despite EpCAM's induction of AXL expression, Bemcentinib, AXL kinase inhibitor, as monotherapy, failed to induce apoptosis (Fig. 4F). This result is consistent with prior findings in KPL tumors, where Bemcentinib monotherapy was ineffective and led to tumor progression, and the outcome was reversed by combination therapy [58].

Our results were closely linked to translational studies. Despite an overall inverse correlation between AXL and EpCAM across large cohorts, we identified a distinct and clinically aggressive subset of NSCLC tumors (25–50%) that co‐expressed both markers. In the tumor cohort, high AXL and EpCAM expression formed a gene‐enriched cluster with significantly higher disease recurrence (40%) and poorer overall survival (Fig. 6F,G), underscoring the prognostic relevance of the EpCAM‐AXL axis. The intriguing observation is that in a subset of tumors, particularly in acquired resistance settings, EpCAM expression may *reappear* or be maintained alongside AXL overexpression, presenting a “hybrid” E/M phenotype or an atypical EMT state relevant to drug resistance. This “switching” or sustained expression is critical to our findings. Consequently, we propose this axis as a powerful dual‐biomarker candidate, potentially leveraging liquid biopsy for circulating sEpCAM and sAXL levels to identify high‐risk patients and guide therapy selection. Furthermore, the 24‐gene signature derived from the AXL/EpCAM co‐expression (Fig. S4B) revealed broad functional involvement, which is crucial for tumor biology beyond direct drug resistance. This includes diverse pathways such as p53‐mediated tumor suppression, integrin‐dependent cell adhesion and migration, and Rho GTPase‐regulated cytoskeletal dynamics. In addition, our Gene Ontology analysis also points to platelet involvement, a finding highly relevant given that platelets play a role in tumor immune evasion [52, 53].

The ubiquitin‐proteasome system (UPS) is the principal mechanism by which cells selectively remove damaged, misfolded, or regulatory proteins, making its inhibition a potent anticancer strategy [59]. The centrality of the proteasome to protein recycling and its key role in AXL degradation shown here underscores its therapeutic relevance. Although proteasome inhibitors primarily block NF‐κB, they also induce the accumulation of p53, p21, and p27; reduce BCL‐2 family members; suppress XIAP; induce ER stress; and inhibit transcription factors, collectively promoting apoptotic cell death.

The ubiquitin‐proteasome pathway is vital for cell survival and proliferation and is thus a promising cancer target. Bortezomib, a 26S proteasome inhibitor, exerts anticancer effects on NSCLC by inhibiting cell growth, promoting apoptosis, and curbing angiogenesis. Recent studies have highlighted proteasome inhibition as a promising therapeutic strategy for NSCLC [60]. Although these agents have shown modest single‐agent activity in solid tumors [61], they have demonstrated synergistic potential in combination with other chemotherapeutic agents, including EGFR inhibitors [62]. Preclinical and early clinical evidence indicate their efficacy across various molecular subtypes, with individual agents differing in potency, tolerability, and mechanisms of action [63, 64, 65, 66]. From a therapeutic point of view, first‐generation inhibitors, such as bortezomib, may cause side effects, such as peripheral neuropathy; however, next‐generation agents, such as Carfilzomib or Ixazomib, show a reduced neuropathy risk [67, 68, 69]. Our data demonstrate that EpCAM‐mediated AXL expression was suppressed by Bortezomib therapy. Thus, EpCAM‐AXL co‐expression defines a therapeutic opportunity in a subset of NSCLC patients, warranting the evaluation of targeted strategies such as proteasome inhibition or EpCAM antibody therapy. While these preliminary findings are compelling and shed light on potential new regulatory pathways influenced by the EpCAM‐AXL axis, extensive validation in appropriate preclinical models and patient cohorts is crucial to fully elucidate the clinical implications and confirm the predictive and prognostic power of this multifaceted gene signature.

The broader context of ADAM17 generating sEpCAM is also critical. ADAM17‐mediated shedding drives pro‐tumorigenic signaling in NSCLC by releasing growth factors and ecto‐domains that engage receptor tyrosine kinases and remodel the tumor microenvironment [14, 15, 70]. As a key membrane protease, ADAM17 cleaves membrane‐bound precursors such as EGFR ligands and TNF‐α, liberating active soluble ligands that promote proliferation, survival, invasion, angiogenesis, and immune suppression [13, 70]. ADAM17‐dependent generation of sEpCAM (or EpCAM‐265), which converts EpCAM from a passive marker into an active signaling molecule, is of particular significance [16]. By “weaponizing” EpCAM and simultaneously shedding other cancer‐relevant substrates, elevated ADAM17 activity can fuel oncogenic cascades, correlate with aggressive disease and poor prognosis, and highlight ADAM17 and its substrate‐processing pathways as attractive therapeutic targets. Our data further support EpCAM‐AXL findings within the broader AXL‐tumor microenvironment, revealing AXL's prevalence on tumor cells, T cells, macrophages, natural killer (NK) cells, fibroblasts, and other immune‐stromal cells [11].

Although our findings offer substantial new insights, certain limitations of this study warrant consideration. Analysis of EpCAM heterogeneity relies on public datasets and prior reports, and validation in larger, prospectively collected cohorts with detailed clinical annotation is required. Xenograft‐derived cells provide a useful tumor microenvironment but may not fully recapitulate human tumor complexity; therefore, follow‐up studies in more representative *in vivo* models, such as patient‐derived xenografts (PDXs), are crucial. Mechanistic insights into sEpCAM‐mediated AXL stabilization are predominantly derived from the A549 cell line and bioinformatics analyses. Further investigation across various NSCLC subtypes is needed to confirm the broader relevance of the AXL‐EpCAM axis, particularly its potentially heightened role in EGFR‐mutant tumors. Despite these limitations, the AXL‐EpCAM axis gene signature and its novel connections to p53, integrin, Rho GTPase‐mediated signaling, and unexpected implications for platelet biology and immune evasion open new avenues for research.

## 5 Conclusion

In conclusion, our study identifies a significant role for EpCAM in NSCLC, particularly during the onset of drug resistance. We propose that the combined assessment of EpCAM‐AXL expression and its ADAM17‐mediated cleavage offers a potential liquid biopsy approach. This would allow for the identification of the EpCAM‐AXL axis as both a driver of drug resistance and a promising therapeutic target. These findings advance our understanding of NSCLC progression and treatment failure, thereby supporting the rationale for proteasome inhibition or EpCAM antibody therapy in patients exhibiting EpCAM‐AXL co‐expression.

## Conflict of interest

The authors declare no conflict of interest.

## Author contributions

AG‐A contributed to data acquisition, formal analysis, validation, visualization, writing, review, and editing. MS contributed to methodology and validation. TPF contributed to review & editing. MAS contributed to resources and review. RMB contributed to resources, review, and supervision. NVS contributed to conceptualization, funding acquisition, supervision, data acquisition, formal analysis, validation, visualization, writing, review, and editing.

## Supporting information


**Fig. S1.** Characterization of EpCAM expression and its relationship with AXL, E‐Cadherin, and Vimentin.
**Fig. S2.** Expression of EpCAM and sEpCAM (soluble EpCAM) confers drug resistance in A549 cells.
**Fig. S3.** AXL re‐expression restores Crizotinib resistance in EpCAM‐depleted cells.
**Fig. S4.** Development of an EpCAM‐AXL expression subgroup gene signature to guide patient therapy.

**Table S1.** Drugs used for the study.
**Table S2.** Real‐Time PCR Primers used for qPCR.
**Table S3.** Antibodies used for the study.

## Data Availability

The original contributions of this study are included in the article/Supporting Information. Further inquiries can be directed to the corresponding authors.
