# Supplementary material for: Targeting the EpCAM‐AXL axis to overcome drug resistance in lung cancer
Source: Mol Oncol. 2026 Oct 6:10.1002/1878-0261.70342. Online ahead of print. doi: 10.1002/1878-0261.70342 (PMC13639404; doi:10.1002/1878-0261.70342)
Supplement: Supplementary file 1 [file MOL2-9999-0-s001.pdf]

Supplementary Table-1 Drugs

Name	Drug Use	IC50	Provider
Afatinib	inhibits EGFR (WT,L858R-T790M, HI	10nm	LC Labs
AICAR	AMPK activator	>200uM	LC Labs
Alvespimycin (17-DMAG)	HSP90 inhibitor	60nm	Selleck Chemi
Bafilomycin A1	H-ATPase Inhibitor	1nm	Sigma
Berberine Chloride	Caspase3-8, JNK P38 Apoptosis acti	14nm	Sigma
Bemcentinib	AXL inhibitor	15nm	Selleck Chemi
BI-2536	Polo like kinase inhibitor	10nm	Selleck Chemi
Bortezomib	Proteasome Inhibitor	10nm	LC Labs
Camptothecin	Topoisomerase Inhibitor	0.7um	Sigma
Cabozantinib	inhibits c-Met, Ret, Kit, Flt-1/3/4, T	10nm	Selleck Chemi
Celecoxib	selective COX-2 inhibitor	50nm	LC Lab
Cisplatin	inhibit DNA synthesis	50um	Sigma
Crizotinib	inhibitor of c-Met ALK, ROS	11-24nm	LC Labs
Curcumin	HDAC	25um	Sigma
Dasatinib	Abl,Src,c-kit Inhibitor	80nm	SellekChem
Doxorubicin	inhibits topoisomerase II	0.8um	LC Labs
Erlotinib	EGFR inhibitor	2nm	LC Labs
Etoposide	topoisomerase II inhibition	250nm	Sigma
Ganetespib (STA-9090)	HSP90 inhibitor	5 nM	Selleck Chemi
Gefitinib	EGFR inhibitor	50nm	Selleck Chemi
GSK2606414	PERK inhibitor	0.4nm	Selleck Chemi
KC7F2	HIF-1α inhibitor	20um	Tocris Biosciences
Lapatinib	EGFR and ErbB2 inhibitor	10nm	LC Labs
Lonafarnib (SCH66336)	H-K-N Ras Inhibitor	5nM	Selleck Chemi
LY294002	PI3Kα/δ/β inhibitor	0.5um	LC Labs
Menadione	inhibitor of Cdc25 phosphatase and mitochondri		Sigma
MG132	Proteasome inhibitor	0.1-1.2um	Selleck Chemi
Nocodazole	inhibitor of microtubule polymerizatio	0.5um	Sigma
Nutlin-3A	inhibits the p53/MDM2 interaction	90nm	Sigma
Osimertinib (AZD9291)	mutant-selective EGFR inhibitor	4-500nm	LC Labs
Paclitaxel (Taxol)	both mitotic arrest and apoptotic cell	50nm	LC Labs
Piperine	traditional medicine	50um	Aldrich
Quercetin	PI3K inhibitor	10um	Sigma
Rapamycin	mTOR inhibitor	1nm	LC Labs
Resvertrol	anti-cancer, anti-inflammatory	2um	Oakwood Chemical
Ruxolitinib	JAK1/2 inhibitor	4nm	LC Labs
SB203580	p38 MAPK inhibitor, PKB	0.5-5um	LC Labs
Sotorasib (AMG510)	KRAS G12C Inhibitor	50 nM	Selleck Chemi
SP600125	JNK Inhibitor	100nm	LC Labs
Sunitinib malate	CCR5 antagonist, VGFR2,PDGFRB	1nm	Tocris Biosciences
Tamoxifen	blocks estrogen action	25nm	Sigma
Trametinib	potent MEK1/2 inhibitor	2nm	LC Labs
U0126	selective inhibitor of MEK1/2	100nm	LC Labs
Y-27632	ROCK1/2 inhibitor	100-300nr	LC Labs

Supplementary Table 2. Real-Time PCR Primers used for qPCR.

Primer Name	Sequence F	Sequence R
EpCAM	CTGCCAAATGTTTGGTGATG	ACGCGTTGTGATCTCCTTCT
E-cadherin	CCTGGGACTCCACCTACAGA	GTGTATGTGGCAATGCGTTC
Vimentin	CAGGCTCAGATTCAGGAACAG	CAGAGAGGTCAGCAAACTTGG
AXL	TGTTTGGTGTTTCTGGGACA	TCGCAGGAGAAAGAGGATGT
MAPK13	GTGCAGAAGCTGAACGACAA	TCAAAGAAGGGATGGGTGAG
18SrRNA	TCAAGAACGAAAGTCGGAGG	GGACATCTAAGGGCATCACA

Supplementary Table 3: Antibodies used for the study

Antibody	Vendor	Cat #
EpCAM	Santa-Cruz Biotech	Sc-25308
E-Cadherin	Santa-Cruz Biotech	Sc-8426
EGFR	Santa-Cruz Biotech	Sc-373748
AXL	Santa-Cruz Biotech	Sc-166269
pAXL-Y702	Cell Signaling	5724
Flag	Sigma	F3165
Vimentin	Cell Signaling	3932
PTEN	Cell Signaling	9552
P53	Santa-Cruz Biotech	Sc-126
c-Met	Santa-Cruz Biotech	Sc-8307
p-EGFR	Cell Signaling	2244
p-ERK1/2	Cell Signaling	9101
ERK1/2	Cell Signaling	9102
pAKT-S473	Cell Signaling	4051
AKT	Cell Signaling	9272
pSTAT3-Y705	Cell Signaling	9145
Beta-Actin	Santa-Cruz Biotech	Sc-47778
Caspase-3	Cell Signaling	9662
PARP1	Cell Signaling	9542
BIM	Santa-Cruz Biotech	Sc-11425
GAPDH	Santa-Cruz Biotech	Sc-47724
UB	Santa-Cruz Biotech	Sc-8017
