# Supplementary material for: Targeting the EpCAM‐AXL axis to overcome drug resistance in lung cancer
Source: Mol Oncol. 2026 Oct 6:10.1002/1878-0261.70342. Online ahead of print. doi: 10.1002/1878-0261.70342 (PMC13639404; doi:10.1002/1878-0261.70342)
Supplement: Supplementary file 2 [file MOL2-9999-0-s002.pdf]

Supplementary figure 1.

A.

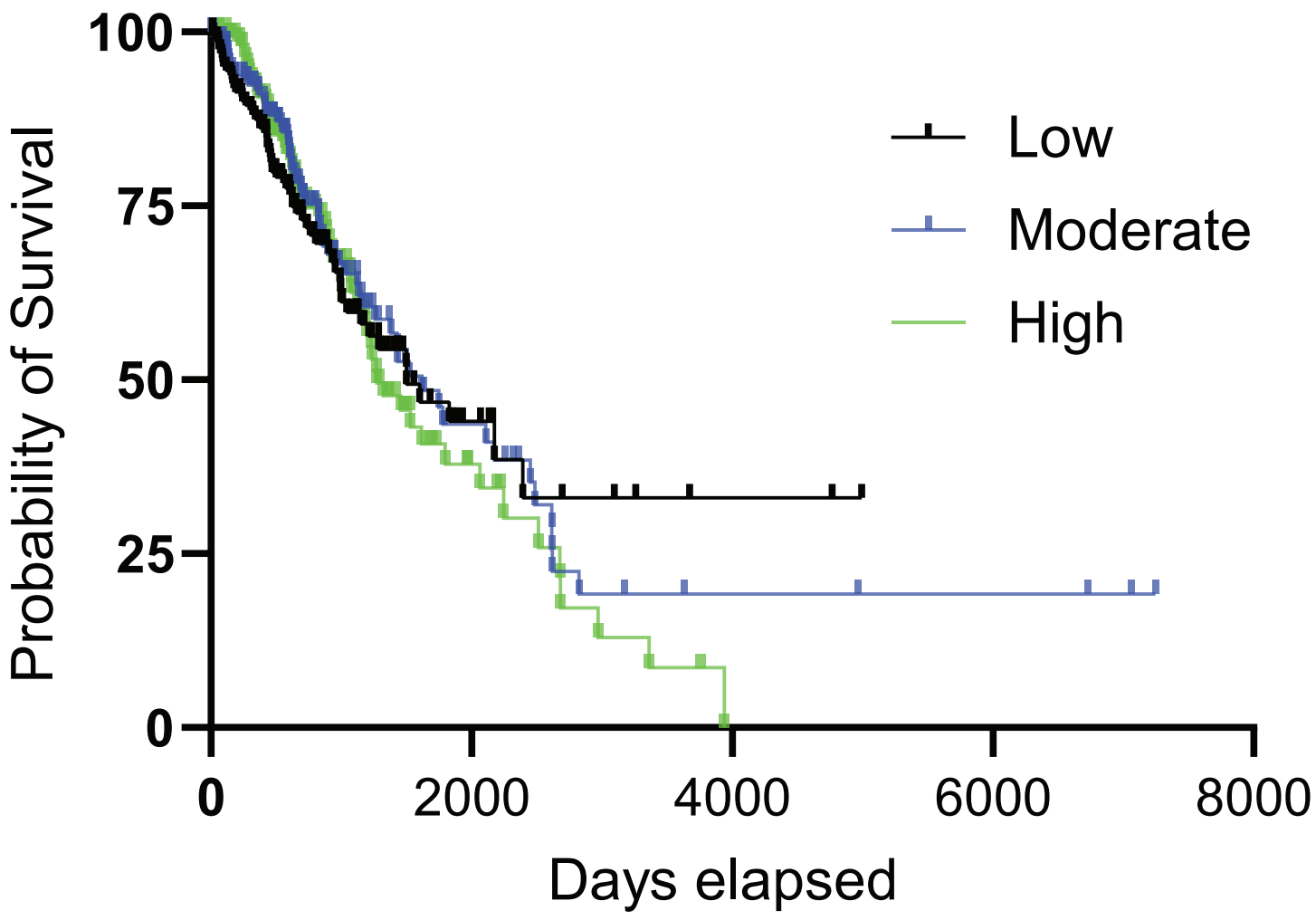

B.

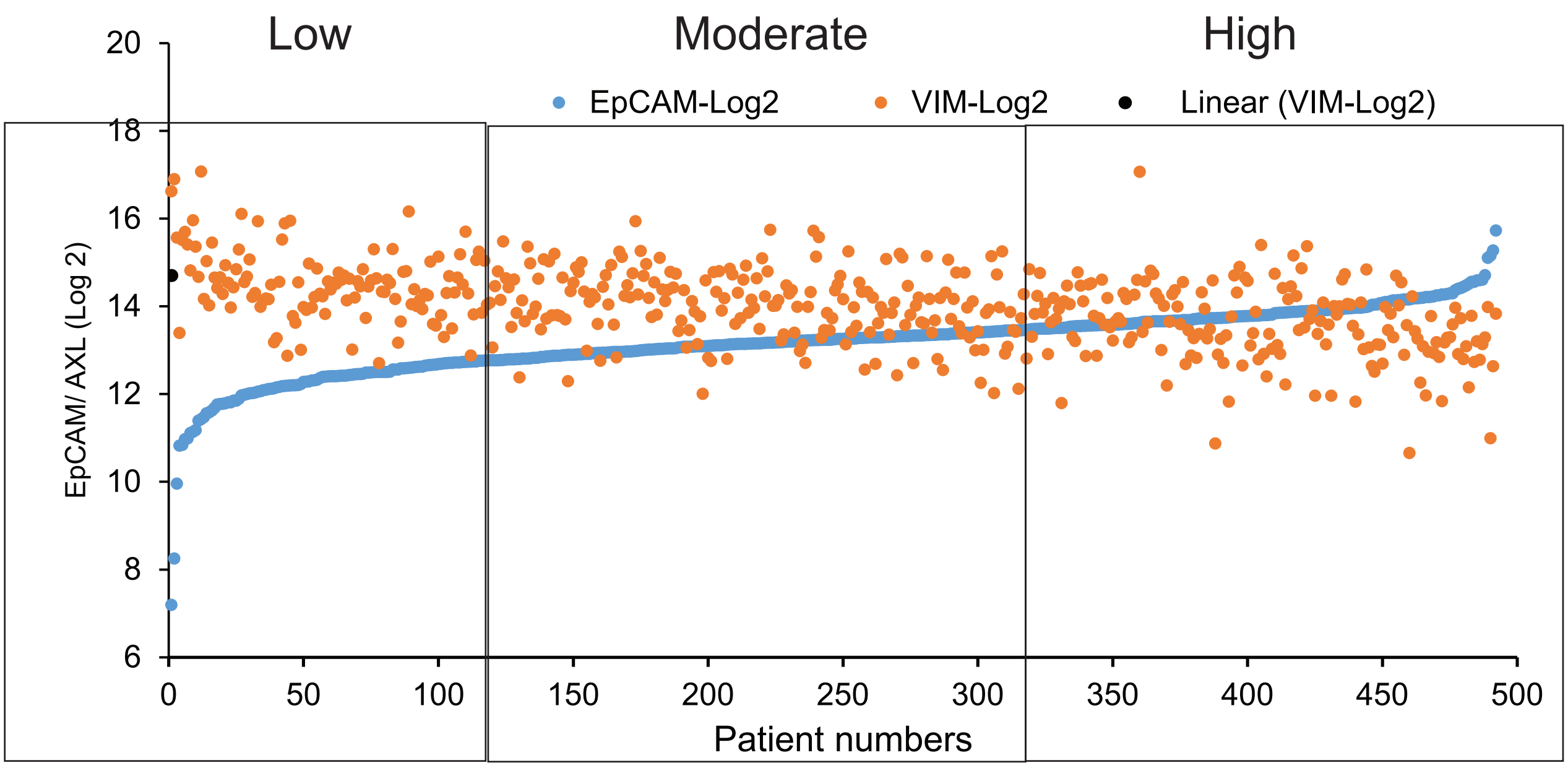

C.

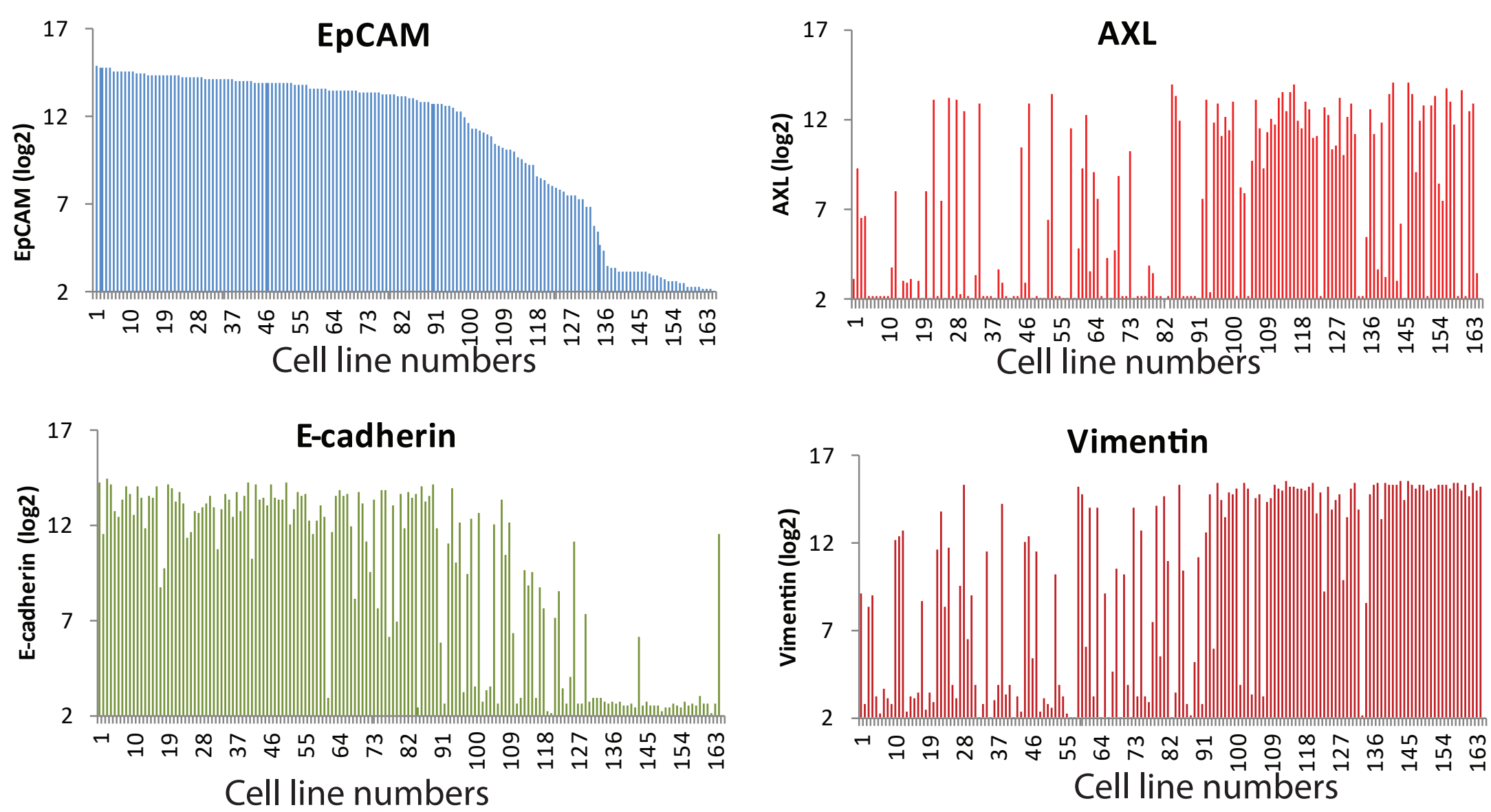

Supplementary figure 1.

D.

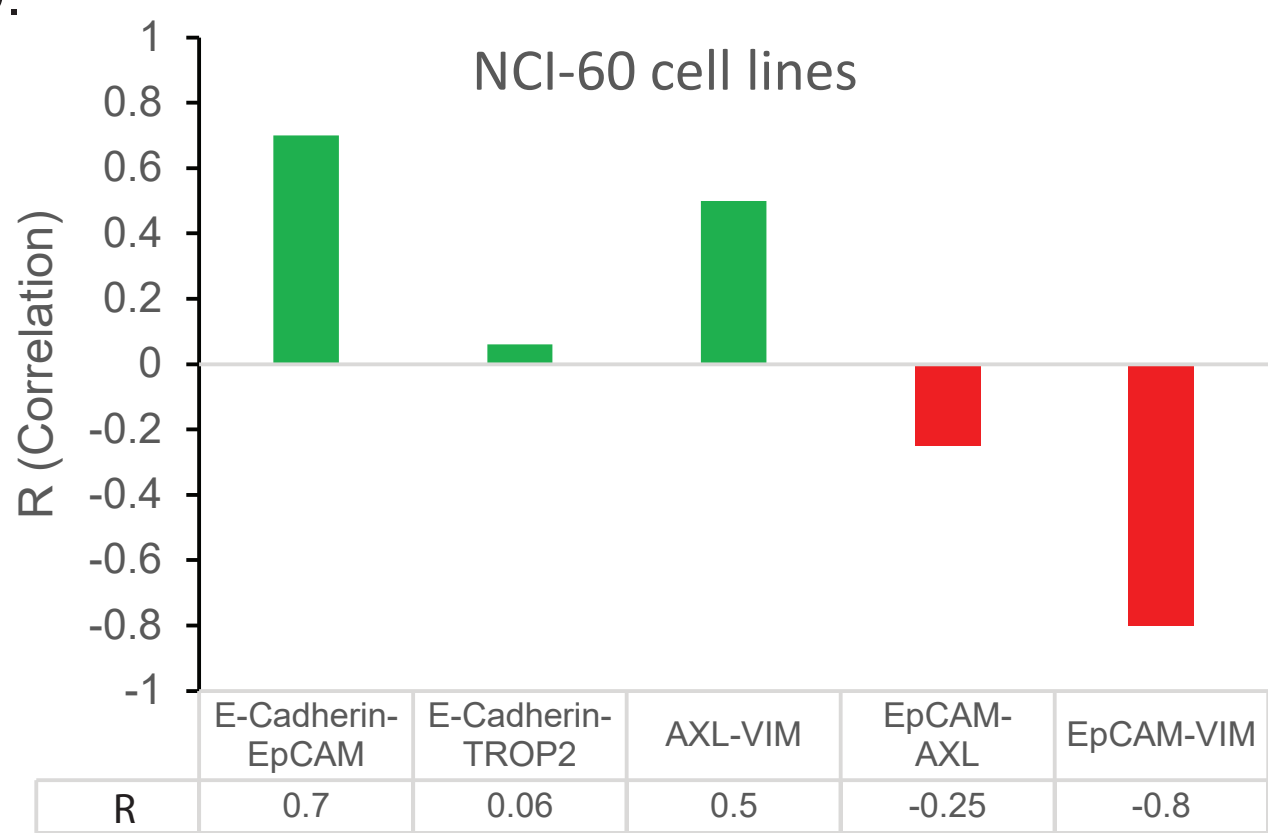

E.

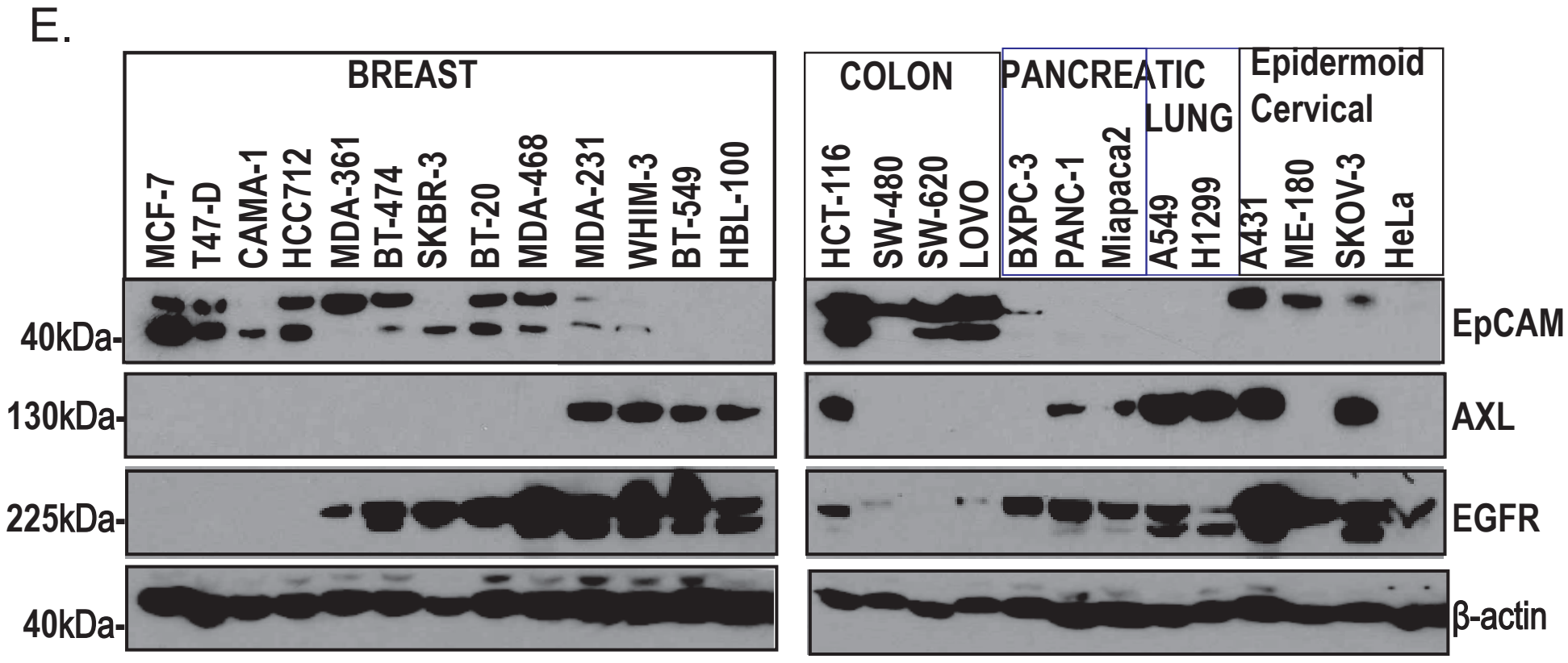

Supplementary Figure 1: Characterization of EpCAM expression and its relationship with AXL, E-Cadherin, and Vimentin. (**A**) Clinical relevance of EpCAM expression: Kaplan-Meier survival analysis in TCGA Lung Adenocarcinoma (LUAD) patients. Patients with higher EpCAM expression demonstrated significantly poorer survival outcomes, indicating an inverse correlation. (**B**) EpCAM and Vimentin co-expression in TCGA LUAD: Scatter plot showing the strong positive correlation (R=0.8, P=0.012) between EpCAM and Vimentin expression, supporting their utility in defining distinct expression subgroups. **(C**) Gene expression landscape in epithelial cell lines: Analysis of AXL, E-Cadherin, and Vimentin mRNA levels across 166 Pfizer epithelial cell lines (GSE34211), with lines ordered by decreasing EpCAM expression to illustrate co-expression patterns. (**D**) Correlates of EpCAM expression in the NCI-60 panel: Gene expression correlation analysis (GSE5846) demonstrating a robust positive correlation of EpCAM with E-Cadherin (R= 0.7) and a strong negative correlation with Vimentin (R= -0.8). (**E**) Protein expression validation: Western blot showing protein levels of EpCAM in a panel of 26 cancer cell lines derived from breast, colon, pancreas, lung, and epidermoid tumors. EpCAM low, moderate cells expressed AXL, and EGFR. Experiments were repeated three times.

Supplementary Figure 2.

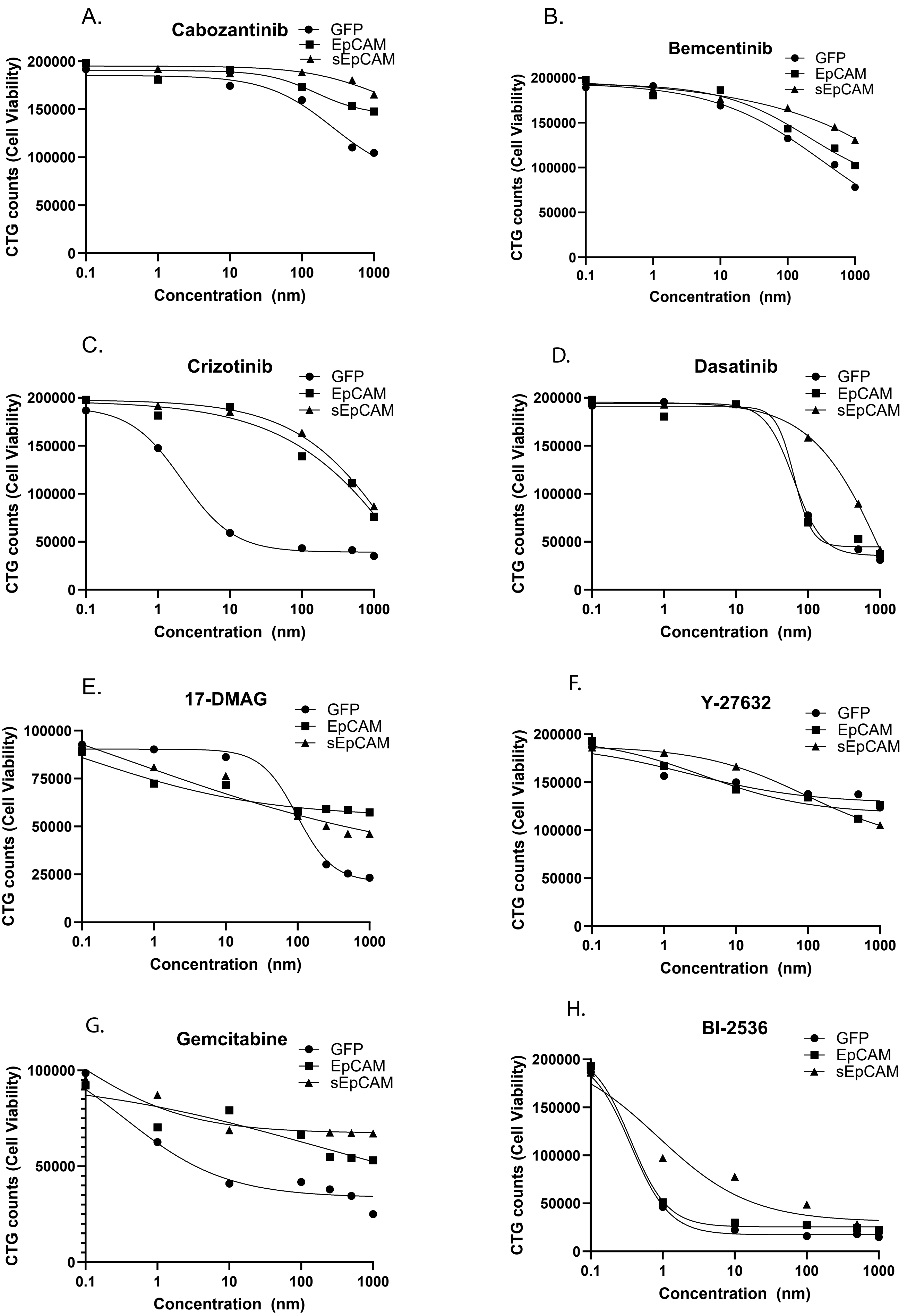

Supplementary Figure 2: Expression of EpCAM and sEpCAM (soluble EpCAM) confers drug resistance in A549 cells. (A-H) Dose-response curves illustrating the impact of EpCAM variants on cell survival following drug treatment. A549-GFP (control), A549-EpCAM-314F and A549-sEpCAM-265Fcells were treated with a range of concentrations (0.1 nM to 1000 nM) of the specified drugs for 72 hours. Cell viability was then determined by CellTiter-Glo assay. Specific drugs tested include: (**A**) Cabozantinib, (**B**) Bemcentinib, (**C**) Crizotinib, (**D**) Dasatinib, (**E)** 17-DMAG, (**F**) Y-27632, (**G**) Gemcitabine, and (**H**) BI-2536.

Supplementary Figure 3.

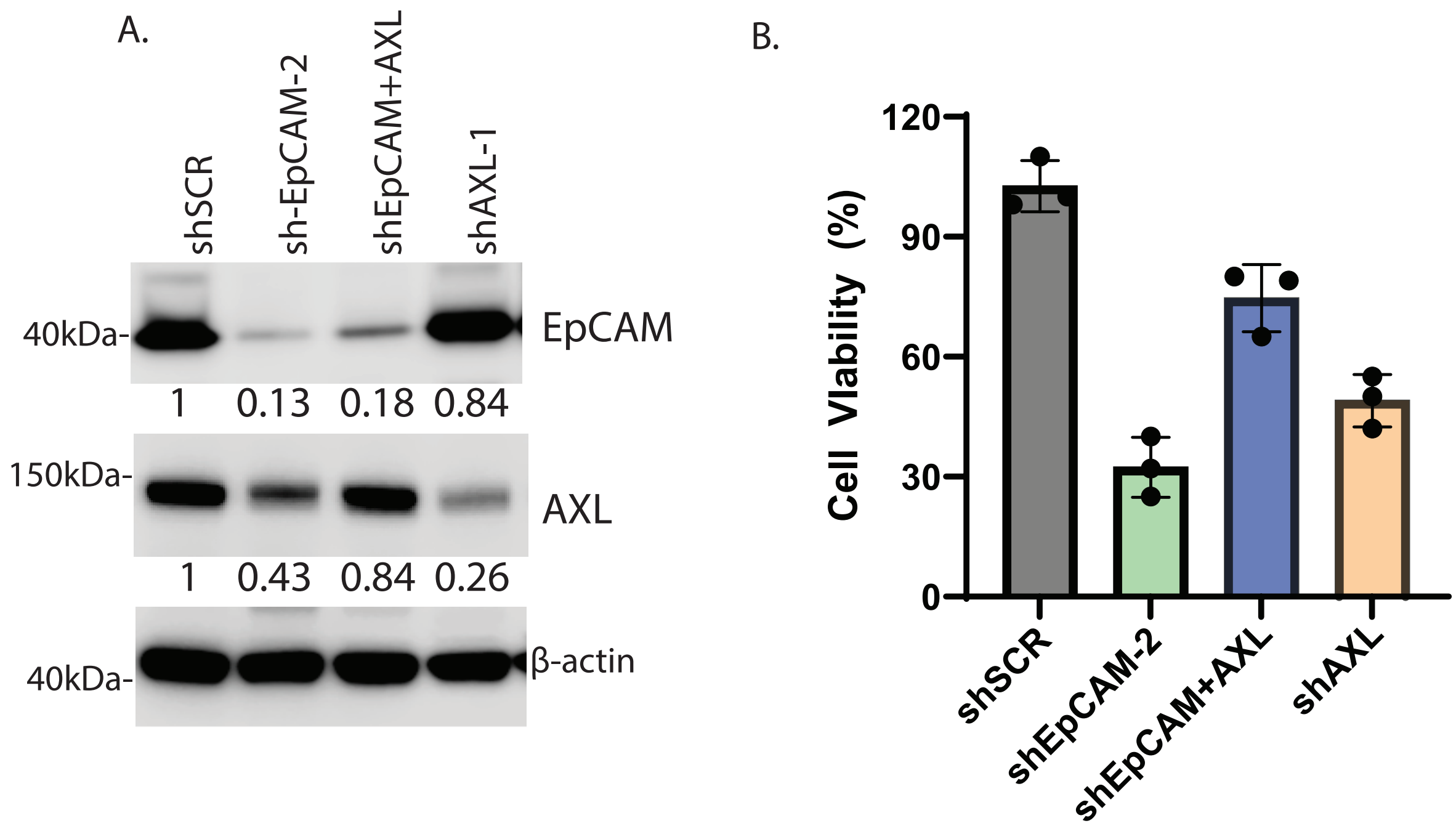

Supplementary Figure 3. AXL re-expression restores Crizotinib resistance in EpCAM-depleted cells. A549-265F cells with stable EpCAM knockdown (shEpCAM-2) were transiently transfected with either a pCMV-AXL expression vector to re-express AXL or an empty vector control. **(A)** Validation of EpCAM and AXL expression. Lysates from cells were subjected to immunoblot analysis for EpCAM and AXL, with beta-actin serving as a loading control. **(B)** AXL-mediated rescue of Crizotinib resistance. Following 24 hours of transfection, cells (including control, the AXL-rescued shEpCAM-2 cells, and stable shAXL knockdown cells for comparison) were treated with Crizotinib (100nm) for an additional 48 hours. Cell viability was subsequently assessed using a CellTiter-Glo assay. Data represent the mean ± SEM from three independent experiments. These results demonstrate that the Crizotinib sensitivity induced by EpCAM ablation is dependent on AXL expression. All experiments were repeated three times.

Supplementary Figure 4.

A. GSE4824 (NSCLC cell lines)

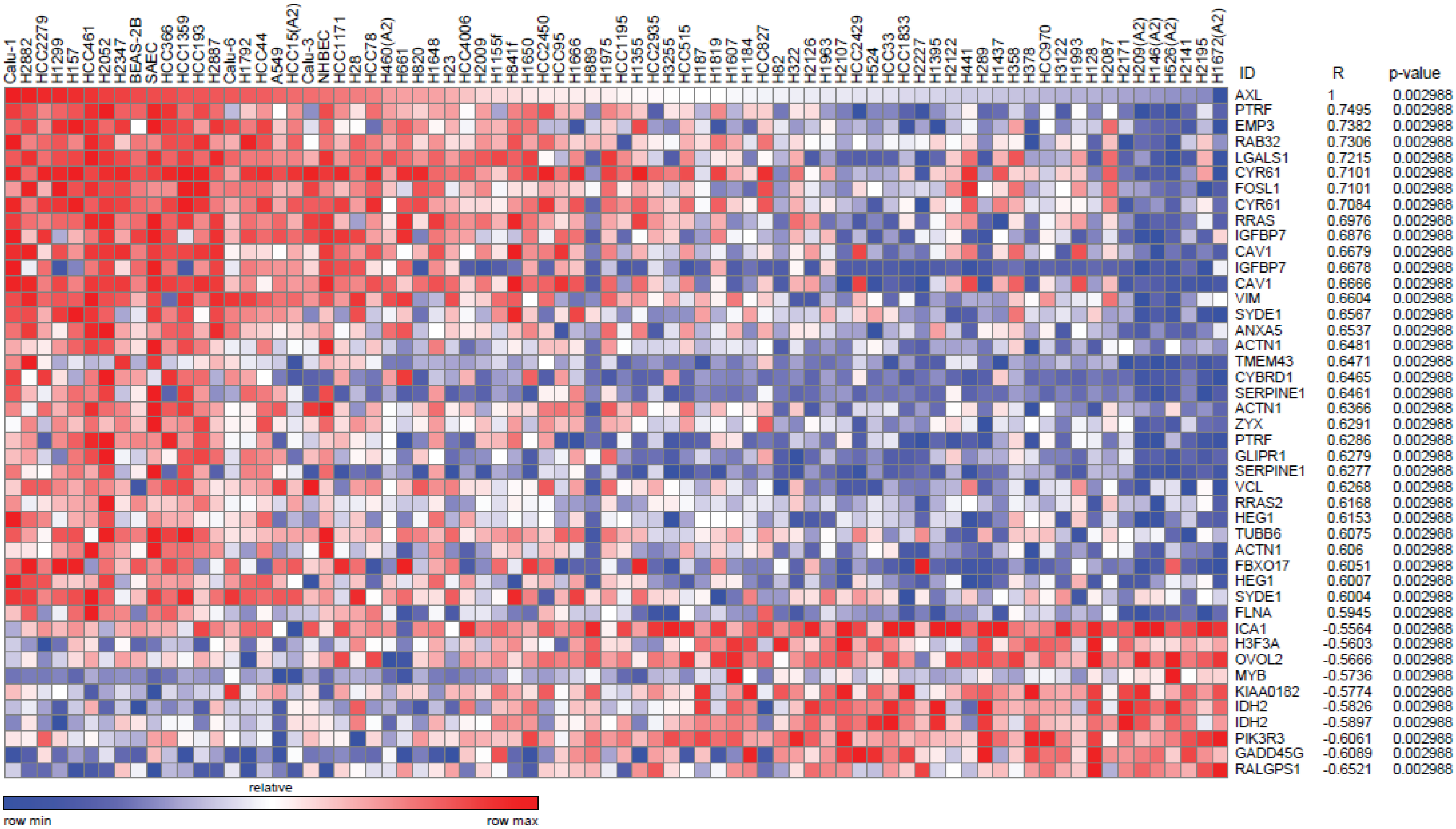

B.

GSE4824
GSE32989

Gene	R
AXL	1
EMP3	0.738229
RAB32	0.73064
LGALS1	0.721527
CYR61	0.710107
FOSL1	0.710061
RRAS	0.697597
IGFBP7	0.687625
CAV1	0.666622
VIM	0.660413
ANXA5	0.65367
ACTN1	0.648111
TMEM43	0.647125
CYBRD1	0.646532
SERPINE1	0.646061
ZYX	0.629056
PTRF	0.628644
GLIPR1	0.627891
VCL	0.626797
HEG1	0.615274
TUBB6	0.607535
FBXO17	0.605144
SYDE1	0.600425
FLNA	0.594483
ICA1	-0.55637
H3F3A	-0.56028
OVOL2	-0.56662
MYB	-0.57365
KIAA0182	-0.5774
IDH2	-0.58259
PIK3R3	-0.60608
GADD45G	-0.60892
RALGPS1	-0.65208

C.

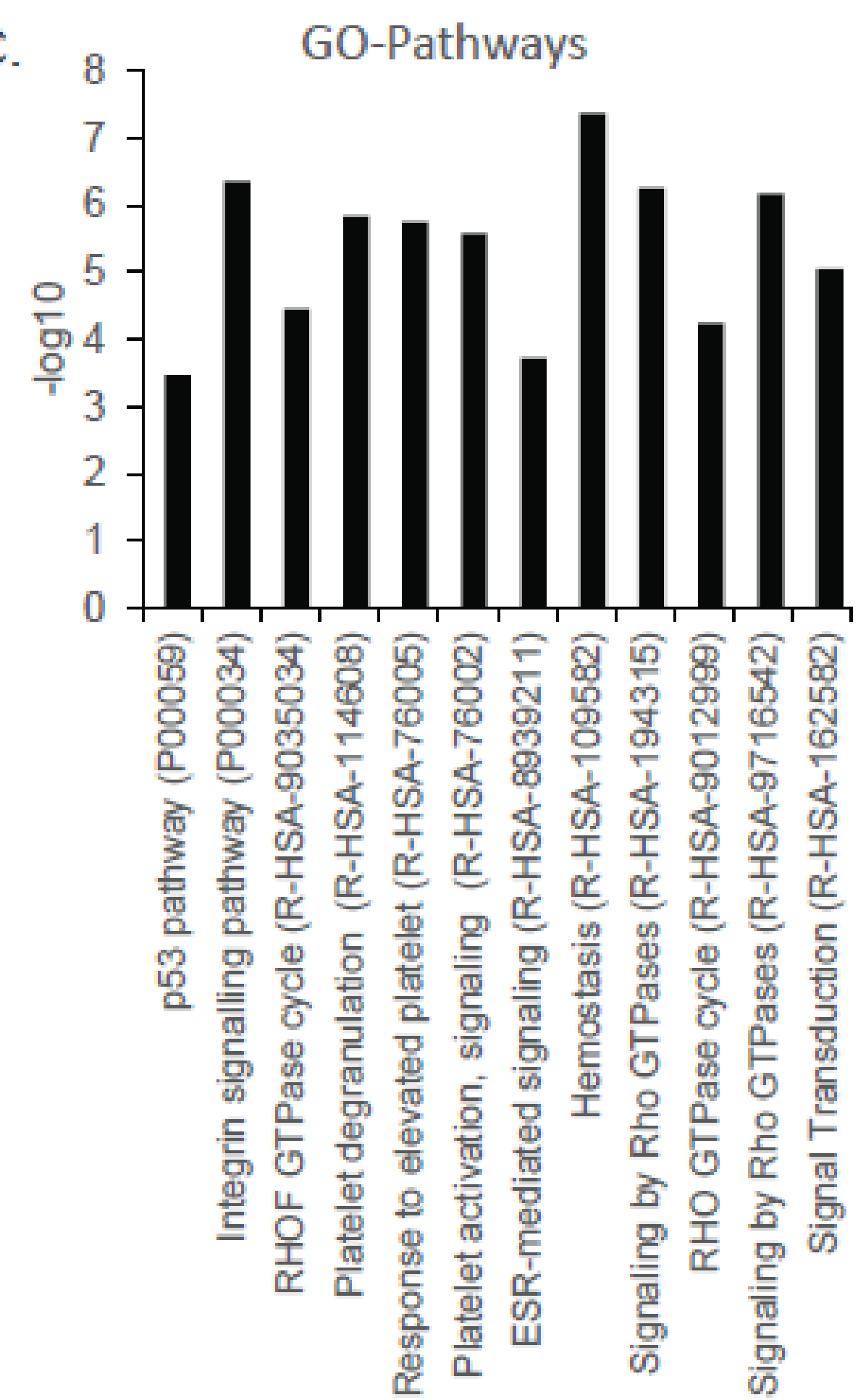

Supplementary Figure 4: Development of an EpCAM-AXL expression subgroup gene signature to guide patient therapy. (A) Correlated genes with AXL and EpCAM in NSCLC. Analysis of NSCLC cell line gene expression data (GSE4824) identified genes whose expression patterns were significantly correlated (both positively and negatively) with those of both AXL and EpCAM. (B) Functional characterization of the EpCAM-AXL gene signature. An independent NSCLC dataset (GSE32989) was employed to generate and refine the EpCAM-AXL axis gene signature. Gene Ontology (GO) analysis of the positively correlated genes within this signature revealed associations with key cancer pathways, including p53, integrin signaling, Rho GTPase activity, and suggests a role in inhibiting immune responses in cancer cells.
